# A Combination of Long-Day Suppressor Genes Contributes to the Northward Expansion of Rice

**DOI:** 10.3389/fpls.2020.00864

**Published:** 2020-06-16

**Authors:** Yongxia Cui, Junrui Wang, Li Feng, Sha Liu, Jiaqi Li, Weihua Qiao, Yue Song, Zongqiong Zhang, Yunlian Cheng, Lifang Zhang, Xiaoming Zheng, Qingwen Yang

**Affiliations:** ^1^National Key Facility for Crop Gene Resources and Genetic Improvement, Department of Center for Crop Germplasm Resources, Institute of Crop Sciences, Chinese Academy of Agricultural Sciences, Beijing, China; ^2^Department of Center for Crop Germplasm Resources, Rice Research Institute, Guangxi Academy of Agricultural Sciences, Nanning, China

**Keywords:** *Oryza sativa*, long-day suppressor genes, domestication, northward expansion, heading date

## Abstract

Growing cultivated rice with a moderate heading date is the key to expanding its cultivation area and maintaining stable yields. The genes that regulate heading date are largely cloned; however, it remains unclear how genetic mutations and their combinations affect the heading date and adaptability of cultivated rice. Here, we report the analysis of genetic variation in eight long-day flowering suppressor genes (*Hd1, DTH8, Ghd7, OsCOL4, DTH7, Hd6, Se5*, and *PhyB*) and the phylogenetic relationship of eight genes. Genetic variations in *DTH8*, *Ghd7*, *Hd1*, *DTH7*, *PhyB*, and *OsCOL4* are correlated with differences in heading date and the correlation between the genetic diversity of *Hd6* and *Se5* and rice heading data are weak. One group of haplotypes of *DTH8*, *Ghd7*, *Hd1*, *DTH7*, *PhyB*, and *OsCOL4* are associated with earlier heading dates and appear to have accumulated during the northward expansion of rice cultivation. A minimum of four group A alleles of *DTH8*, *Ghd7*, *Hd1*, *DTH7*, *PhyB*, and *OsCOL4* are required for the growth of cultivated rice at latitudes above 30°N. This study presents a preliminary investigation of the genetic patterns and adaptation mechanisms of long-day flowering suppressor genes and provides a useful reference for the molecular breeding of rice cultivars for various environments and farming systems.

## Introduction

To make full use of sunlight and temperature resources and to ensure food security, it is necessary to selectively breed rice varieties whose heading dates are suited to the climatic characteristics of different regions (Itoh et al., 2018). The ancestral species of cultivated rice (*Oryza rufipogon* Griff.) is a short-day sensitive plant that grows mainly in low-latitude tropical and subtropical regions. After natural selection and artificial domestication, the geographical distribution of cultivated rice gradually expanded from 45°N to 23°S (Monfreda et al., 2008; Huang et al., 2012), growing under both long-day and short-day conditions. During the expansion process, it was important to cultivate varieties with appropriate heading dates. Therefore, exploring the genetic mechanisms that underlie heading date diversity in cultivated rice is important for understanding its domestication history and the cultivation of eurytopic rice species.

Previous studies have shown that photoperiod is an important factor that affects the heading date of cultivated rice (Shang et al., 2012). To date, many genes in the rice photoperiodic regulatory network have been cloned (Gao et al., 2014). These include Heading date 3a (*Hd3a*) and RICE FLOWERING-LOCUS T 1 (*RFT1*), two florigen genes that act as biological signals to complete the phase transition from vegetative to reproductive growth (Kojima et al., 2002; Komiya et al., 2008). Heading date 1 (*Hd1*) and Early heading date 1 (*Ehd1*) are two hub genes in the photoperiodic regulatory pathway that have different responses to day length. They control the formation of florigen genes by receiving multiple regulatory signals from upstream genes and then influencing heading date (Yano et al., 2000; Wei et al., 2016). In rice, *Hd1* suppresses flowering under long-day conditions by interacted with *Ghd7* to repress the expression of *Ehd1* (Nemoto et al., 2016), but promotes flowering under short-day conditions (Yano et al., 2000). *Ehd1* promotes flowering under both long- and short-day conditions (Wei et al., 2016). Under short-day conditions, *Ehd1* is regulated by multiple upstream genes that include OsGIGANTEA (*OsGI*), Early heading date 2 (*Ehd2*), and MADS BOX GENE 51 (*OsMAD51*) (Matsubara et al., 2008; Ryu et al., 2009). Under long-day conditions, *Ehd1* is directly or indirectly regulated by MADS BOX GENE 50 (*OsMAD50*), Early heading date 2 (*Ehd2*), Early heading date 3 (*Ehd3*), and Early heading date 4 (*Ehd4*). Furthermore, HEADING DATE 6 (*Hd6*), Heading date (QTL)-5 (t) (*DTH8*), Heading date 2 (*DTH7*), CONSTANS-like gene 4 (*OsCOL4*), HEADING DATE 7 (*Ghd7*), PHYTOCHROME B (*PhyB*), and PHOTOSENSITIVITY 5 (*Se5*) also directly or indirectly suppress *Ehd1* expression (Dehesh et al., 1991; Takahashi et al., 2001; Xue et al., 2008; Lee et al., 2010; Wei et al., 2010; Hyuk et al., 2013; Du et al., 2017). In summary, the photoperiodic regulatory network in cultivated rice can be divided into short-day promotion, long-day promotion, and long-day suppression pathways.

Previous research suggests that there is a correlation between genetic diversity of long-day suppressor genes and heading date, and that this diversity was critical for the expansion of cultivated rice production to higher latitudes (Wu et al., 2012; Zheng et al., 2016). Takahashi et al. (2009) found that genetic diversity of *Hd1* was an important reason for flowering date diversity in cultivated rice. They showed that a loss of function of *Hd1* occurred 8,000 to 10,000 years ago and played an important role in the adaptation of rice crops to higher latitudes and in the development of various growing strategies (Takahashi et al., 2009). Wu et al. (2012) reported that genetic variation in *DTH2* was closely related to the northward expansion of Asian cultivated rice (Wu et al., 2012). Koo et al. (2013) found that genetic mutations in *OsPRR37* and *Ghd7* could promote early flowering of cultivated rice during heading under long-day conditions, thereby adapting cultivated rice to higher latitudes (Koo et al., 2013). Gao et al. (2014) showed that different haplotype combinations of the long-day suppressor genes *DTH7*, *DTH8*, and *Ghd7* were closely related to heading date diversity in cultivated rice. Among three-gene haplotype combinations, the combination that included loss of function in *DTH7*, *DTH8*, and *Ghd7* was found mainly in cultivated rice in northern China (Gao et al., 2014).

The aforementioned studies were performed on a limited number of long-day suppressor genes. Less is known about the genetic diversity of long-day suppressor gene pathways and about the accumulation effects of different haplotype combinations of eight long day suppression gene on heading date in cultivated rice. Therefore, in this study, we collected rice cultivars from around the world and analyzed the genetic diversity of eight important long-day suppressor genes: *Hd1*, *DTH8*, *Ghd7*, *OsCOL4*, *DTH7*, *Hd6*, *Se5*, and *PhyB*. We then analyzed the effects of individual haplotypes and haplotype combinations of different long-day suppressor genes on heading date and geographic distribution to better understand the molecular underpinnings of heading date diversity in cultivated rice.

## Materials and Methods

### Plant Materials and Phenotyping

One hundred and forty-eight samples, including 83 from cultivated rice (*Oryza sativa* L.) and 65 from wild rice (*O.nivara* and *O.rufipogon*) were included in the study. Among these accessions, the 83 cultivated rice accessions covered approximately all subgroups and rice planting areas from Northeastern to Southern China and from Central Asia to South Asia and Southeast Asia (**Table S1**, **Figure S1**). The 65 wild rice accessions covered most of tropical and subtropical of Asia from Southern China (28°N) to Indonesia (10°S). Detailed information about the accessions, including names, countries of origin, geographic locations, and subpopulation classifications, is presented in **Table S1**. All seeds were breaked dormancy by keeping at 50°C, 72 h, then soaked for 48 h in water; they were then planted in nutritious soil and raised in wet-bed condition for 23–26 days for reaching the seedling stage. Seedlings were then transplanted into the same agricultural field in Peking (116°13'E, 39°54'N) on May 5, 2016, May 5, 2017, and May 5, 2018, respectively during the summer under natural long-day conditions. Seedlings from each accession were planted in a single row of 10 plants in each of the years. Field management was performed according to typical rice cultivation practices. Heading date was scored for each plant as the number of days from the sowing date to the date at which the first panicle measured 1–2 cm; The mean heading date of 30 plants per accession for the 3 years was calculated (**Table S1**). Of the 83 cultivated rice accessions, 15 did not have heading date data because the seedlings did not survive.

### DNA Isolation, PCR Amplification and Sequencing

Fresh leaves were collected from field-grown rice plants after rice flowering, and genomic DNA was isolated from the fresh tissue by a modified CTAB method and used to amplify the coding regions of *DTH8*, *Ghd7*, *Hd1*, *DTH7*, *PhyB*, *OsCOL4*, *Hd6*, and *Se5*. PCR amplification methods followed standard PCR protocols as described in Zheng and Ge (Zheng and Ge, 2010). The genome sequence of Nipponbare (*O. sativa* ssp. *japonica*) was downloaded from NCBI and used as the reference sequence for primer design (**Figure S2** and **Table S2**). PCR products were sequenced using an ABI 3730 automatic sequencer (Applied Biosystem, Foster City, CA).

### Sequence Analysis

DNA Sequence alignment was performed with ClustalX 2.1 (Larkin et al., 2007) and manually adjusted in BioEdit (http://www.mbio.ncsu.edu). Polymorphic sites were analyzed using DnaSP5.0 (Huelsenbeck and Ronquist, 2001), and insertions/deletions (indels) were included in the analysis. Gaps with a length greater than one were considered to be single mutations. Network trees for the eight genes of interest were constructed using the median-joining model of DnaSP5.0 and Network 4.0 (Bandelt et al., 1999), and divided *DTH8*, *Ghd7*, *Hd1*, *DTH7*, *PhyB*, and *OsCOL4* into two groups (A and B) based on genetic distance.

### Statistical Analysis

To determine whether haplotype in groups A and B of the six long-day suppression genes (*DTH8*, *Ghd7*, *Hd1*, *DTH7*, *PhyB* and *OsCOL4*) differed in heading date, we constructed boxplots of rice heading date data using R version 3.5.2 and used paired *t*-test to assess significant differences. We then investigated differences in frequency of haplotype groups A and B with latitude from 45°N to 23°S by constructing a heatmap in Excel based on rice haplotype coordinate data. Ultimately, to analyze the geographic distribution of group A and group B samples, geographic distribution maps of rice from haplotype groups A and B of each gene were constructed using leaflet function in R based on geographic coordinates. To explore the association between haplotype combinations and climate factors, raw data for Minimum Temperature of Coldest Month were download from the WorldClim Version 2 database (http://worldclim.org/bioclim) at a 30 s (~1 km) resolution. Using sp and rgdal function in R, the data were converted into a climate density map, and haplotype combination data points for *OsCOL4*, *DTH7*, *PhyB*, *Ghd7*, and *DTH8* were overlaid onto the map based on geographic coordinate data. To determine the relationships between haplotype combination and heading date or latitude, we again constructed boxplots in R. The effect of haplotype combination was assessed using the Anova function in R, and mean separations were performed using the LSD function in R (Grunsky, 2002).

## Results

### Genetic Variation

One hundred and forty-eight rice samples were collected from major rice cropping areas, including 83 representative cultivated rice samples and 65 wild rice samples (**Table S1**, **Figure S1**). The coding regions of eight long-day suppressor genes (*DTH8*, *Ghd7*, *Hd1*, *DTH7*, *PhyB*, *OsCOL4*, *Hd6*, and *Se5*8) were sequenced using the primers in **Table S2**. Diagrams of their gene structures are shown in **Figure S2**. Exon numbers ranged from one to ten, with one exon in *DTH8* and 10 in *Hd6*. Sequence lengths ranged from 774 bp to 3516 bp, and 233 SNPs were found among all the sequences (25, 47, 41, 59, 30, 19, 10, and 2 SNPs in *DTH8*, *Ghd7*, *Hd1*, *DTH7*, *PhyB*, *OsCOL4*, *Hd6*, and *Se5*, respectively). Among these SNPs, all mutations in *Se5* were silent mutations that did not affect the coding for amino acids. More than half of the remaining seven genes had SNPs that led to changes in amino acid sequence, and most of the biologically significant mutations were located in the first exon (**Figure S3**). Three, 2, 1, 5, and 1 SNPs that generated premature stop codons were identified in *DTH8*, *DTH7*, *Ghd7*, *Hd1*, and *Hd6*, respectively (**Figure S3**). Three, 4, 2, 6, 3, 1, and 1 indels were identified in *DTH8*, *DTH7*, *Ghd7*, *Hd1*, *PhyB*, *OsCOL4*, and *Hd6*, respectively (**Figure S3**); there were no indels in *Se5* (**Figure S3**). There was greater genetic diversity in *DTH8*, *DTH7*, *Ghd7*, *Hd1*, *PhyB*, and *OsCOL4* than in *Hd6* and *se5*.

### Haplotype Analysis

**Figure 1:**
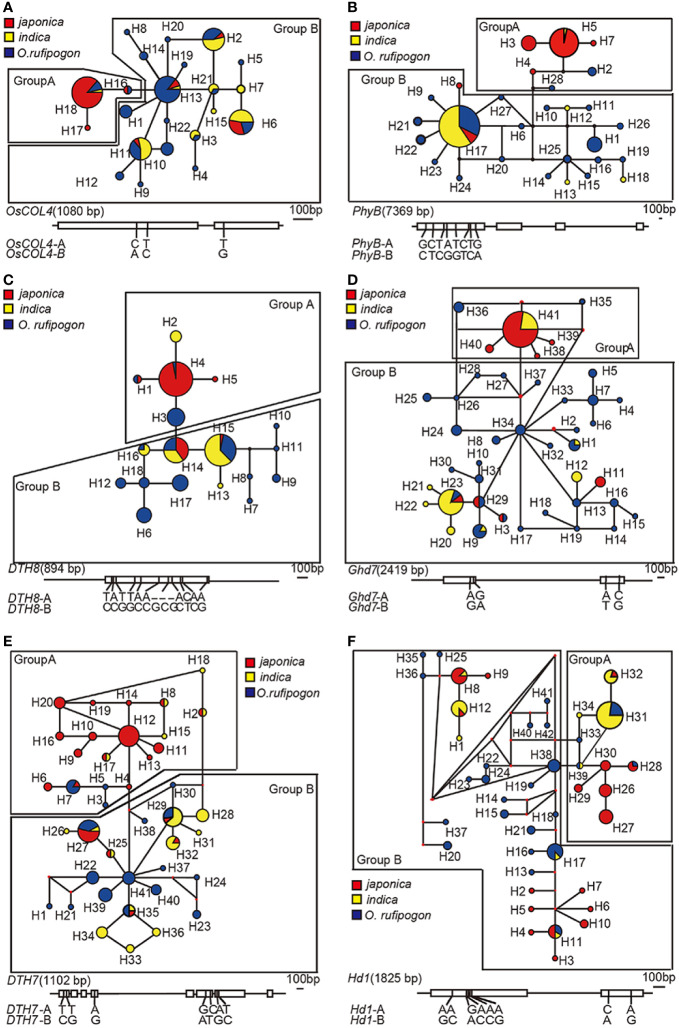
Network trees of six long-day suppressor genes in cultivated rice. **(A)** *OsCOL4*: *H_2, H_6, H_11, H_13*, and *H_18* are high-frequency haplotypes of *OsCOL4*. **(B)** *PhyB*: *H*_5 and *H*_17 are high-frequency haplotypes of *PhyB*. **(C)** *DTH8*: *H*_3, *H*_4, *H*_6, *H*_14, *H*_15, and *H*_17 are high-frequency haplotypes of *DTH8*. **(D)** *Ghd7*: *H*_23 and *H*_41 are high-frequency haplotypes of *Ghd7*. **(E)** *DTH7*: *H*_7, *H*_12, *H*_27, *H*_29, and *H*_32 are high-frequency haplotypes of *DTH7*. **(F)** *Hd1*: *H_8*, *Hap_11, H_12, H_17, H_27* and *H_31* are high-frequency haplotypes of *Hd1*. The size of the circle in each gene's network tree represents the haplotype frequency. Yellow and red represent the two subcultivars of cultivated rice, *indica* and *japonica*, and blue represents *O.rufipogon*. Each gene's network tree corresponds to the SNP on the diagram of long-day suppressor genes below, which illustrates the haplotype-specific SNPs between group A and group B of the long-day suppressor genes.

To assess the genetic relationships among taxa we first calculated the numbers of haplotypes and constructed network tree. Mutation sites of each gene in the 148 samples were analyzed, and 18, 41, 41, 42, 28, 22, 3, and 13 haplotypes were found in *DTH8*, *DTH7*, *Ghd7*, *Hd1*, *PhyB*, *OsCOL4*, *Se5*, and *Hd6*, respectively (**Figure S3**). The number of haplotypes with a frequency greater than 5% was 6, 6, 2, 6, 2, 5, 2, and 2 in *DTH8*, *DTH7*, *Ghd7*, *Hd1*, *PhyB*, *OsCOL4*, *Se5*, and *Hd6*, respectively. Frequencies of these haplotypes ranged from 5.10% to 86% (**Table S3**). Network trees for the eight genes were constructed using the median-joining (MJ) method. Based on the relationship among haplotypes and the haplotype frequency in population, we found that *DTH8*, *DTH7*, *Ghd7*, *Hd1*, *PhyB*, and *OsCOL4* were all centered around high-frequency haplotypes and clearly differentiated into two groups, which we called group A and group B (**Figure 1** and **Figure S3**). In *DTH8*, *DTH7*, *Ghd7*, *PhyB*, and *OsCOL4*, we found that the ancestral haplotypes in group B were mainly composed of *indica* and wild rice, whereas the ancestral haplotypes in group A were mainly composed of *japonica* and wild rice grown in China (**Figure 1**).

**Figure 2:**
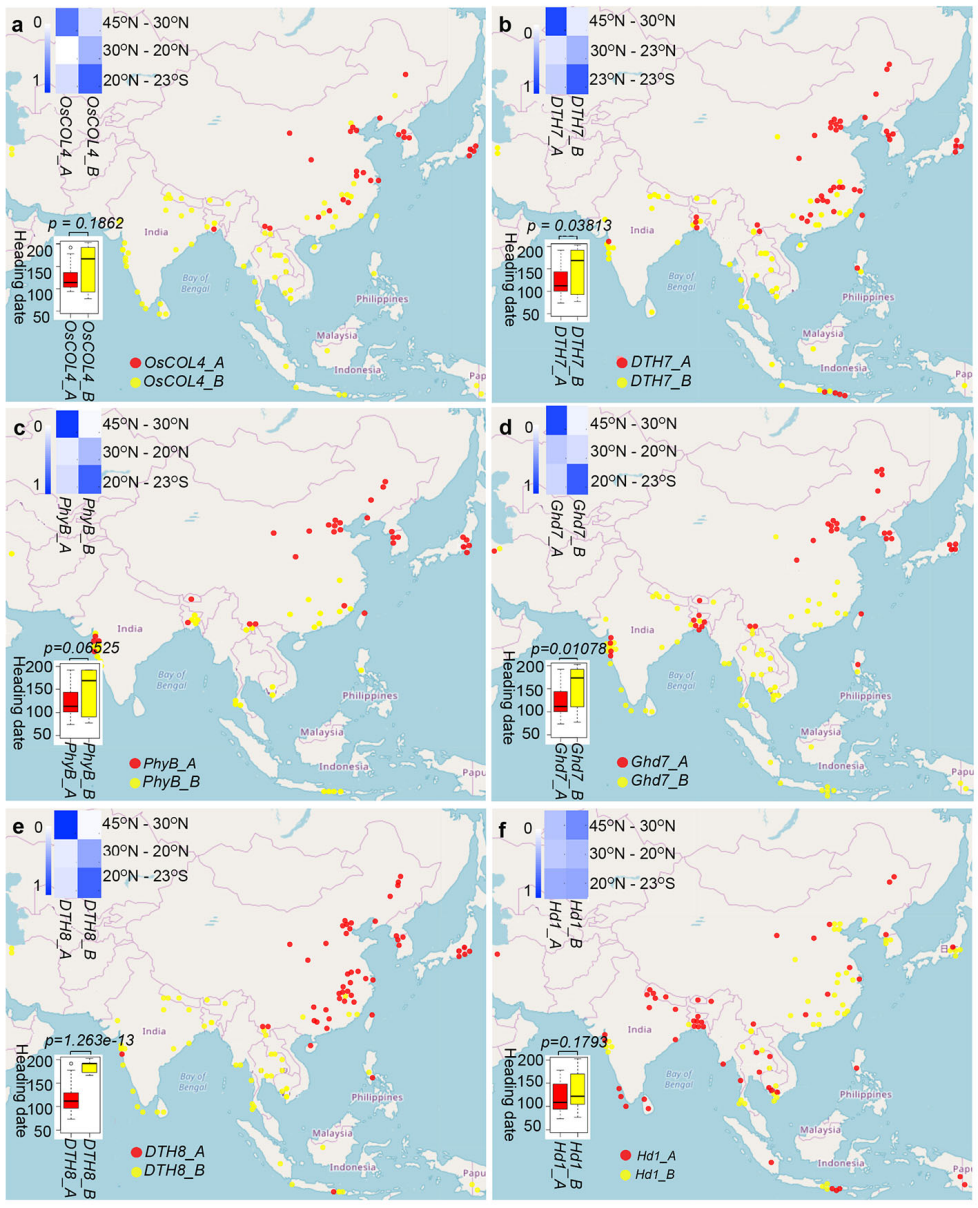
Relationships among haplotypes of six long-day suppressor genes and their heading dates and geographical distribution patterns in cultivated rice. **(A)** *OsCOL4* **(B)** *DTH7* **(C)** *PhyB* **(D)** *Ghd7* **(E)** *DTH8* **(F)** *Hd1*. The boxplots illustrate the correlation between heading date on the *y*-axis and haplotype group (A or B) on the *x*-axis for each of the six genes. Red represents group A haplotypes, and yellow represents group B haplotypes. The blue heatmaps show the frequency of occurrence of each haplotype group at different latitudes. Each cell of the heatmap reports haplotype frequency, and haplotype frequency is accompanied by blue color, with higher haplotype frequency associated with darker blue colorings. The map shows the geographic distribution of haplotype groups for each gene, with red dots representing group A haplotypes and yellow dots representing group B haplotypes.

Additionally, We also analyzed the geographical distribution of samples from both haplotype groups. Group A haplotypes were found at the greatest frequency in cultivated rice from high-latitude regions basically, including Heilongjiang, Jilin, Liaoning, Shanxi, Beijing, and Tianjin in China, as well as from Japan and South Korea. Group B haplotypes were found at the greatest frequency mainly in cultivated rice from the southern regions of China, as well as from tropical and subtropical regions of South and Southeast Asia (**Figures 1** and **2**, **Table S1**). By contrast, although the *Hd1* gene was also centered on high-frequency haplotypes and separated into two groups (i.e. group A and group B), the composition and geographical distribution of subcultivars in groups A and B were different from those of *DTH8*, *DTH7*, *Ghd7*, *PhyB*, and *OsCOL4*. Specifically, both *japonica* and *indica* were found in group A and group B, and cultivated rice species in both groups were distributed from 45°N to 23°S. *Hd6* and *Se5* had only one high-frequency haplotype apiece, and these haplotypes accounted for 81% and 86% of the total haplotypes of the two genes, respectively (**Figure S4**).

### Relationship Between Heading Date of Cultivated Rice and Haplotype of Long-Day Suppressor Genes

To explore the heading date differences between group A and group B. Next, we compared long-day suppressor gene haplotypes with heading dates in cultivated rice. Due to the low genetic diversity of *Se5* and *Hd6*, we only analyzed heading date differences between group A and group B haplotypes for the remaining six genes (**Figure 2**; **Table S4**). For cultivated rice in haplotype group A of *DTH8*, *DTH7*, *Ghd7*, *PhyB*, *OsCOL4*, and *Hd1*, the average heading dates were 116.9, 120.8, 121.1, 121.6, 124.8, and 119.5 days, respectively. By contrast, for cultivated rice in haplotype group B of the same genes, the average heading dates were 185.0, 148.7, 156.5, 147.5, 143.2, and 137.6 days, respectively. Therefore, under long-day conditions, the heading dates of the group A samples were earlier than those of the group B samples. Differences in heading date between group A and group B were statistically significant for *DTH8* (*t* = −10.802, *P* = 1.263e-13, *t*-test), *DTH7* (*t* = −2.1884, *P* = 0.03813, *t*-test), and *Ghd7* (*t* = −2.7768, *P* = 0.01078, *t*-test) (**Figure 2**). The difference between the two groups for *PhyB* was close to significance (*t* = −1.9479, *P* = 0.06525, *t*-test), and differences for the remaining genes were not significant: *OsCOL4* (*t* = −1.3603, *P* = 0.1862, *t*-test) and *Hd1* (*t* = −1.4024, *P* = 0.1793, *t*-test). Taken together, our association studies suggest that group A and group B of *DTH8*, *DTH7*, *Ghd7*, *PhyB*, *OsCOL4*, and *Hd1* are different in terms of heading date regulation. We have added 10 random sequences (Zhu et al., 2007; Wu et al., 2012). Based on these neutral sequence data, congruence between data sets was evaluated using the partition homogeneity test (Farris et al., 1995), as implemented in PAUP with 1,000 replicates, random taxon addition, and one tree saved per replicate. There was signiﬁcant incongruence between any gene (*p*=0.0017 for *DTH8*, *p*=0.024 for *Ghd7*, *p*=0.0033 for *Hd1*, *p*=0.0042 for *DTH7*, *p*=0.019 for *PhyB*, *p*=0.0063 for *OsCOL4*). The resulting *p*-value was used to determine whether the data sets had signiﬁcant incongruence.

### Geographical Evolution Models of Long-Day Suppressor Genes

**Figure 3:**
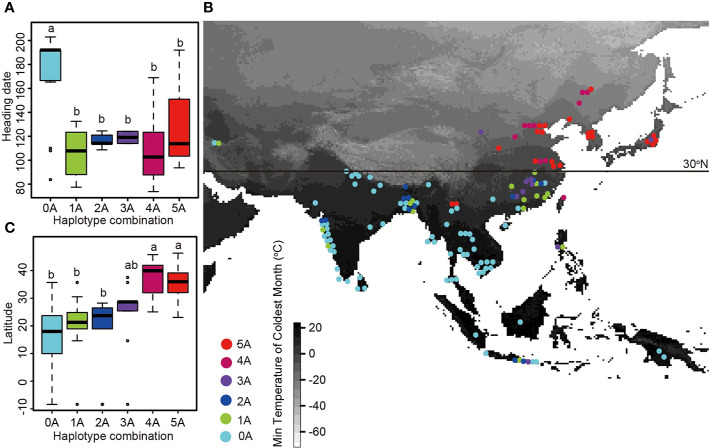
The geographical distribution of haplotype combinations and the relationship between haplotype combination and heading date. **(A)** Boxplots of heading dates for samples with different haplotype combinations of five long-day suppressor genes. Haplotype combinations containing between zero and five group A haplotypes are shown on the *x*-axis. **(B)** Geographical distribution of haplotype combinations of five long-day suppressor genes. Haplotype combinations containing between zero and five group A haplotypes are indicated by dots of different colors. The map is shaded to indicate the minimum temperature of the coldest month, with lower minimum temperatures in white and higher minimum temperatures in black. **(C)** Boxplots of latitude data for samples with different haplotype combinations of five long-day suppressor genes.

Generally, a single gene does not fully explain the genetic basis of different cultivated rice to adapt to different ecological regions. So we next analyzed the geographical distribution of haplotype combinations of the long-day suppressor genes (**Figure 3**, **Table S1**). Because the composition and geographical distribution of *Hd1* subcultivars in group A and group B were different from those of the other genes, we only analyzed the geographical distribution of haplotype combinations of the five long-day suppressor genes for group A and group B haplotypes in *DTH8*, *DTH7*, *Ghd7*, *PhyB*, and *OsCOL4* (**Figure 2**, **Table S1**). Twenty-one samples contained all five group A haplotypes and accounted for 14.2% of all individuals. These samples were composed of *japonica* and were distributed in Japan, South Korea, the Chinese region north to the latitude of 30 degree (Zhejiang, Shanghai, Anhui, Jiangsu, Tianjin, and Shanxi), the high latitudes of Yunnan Province, etc. The average heading date of these samples was 127.12 days. Eleven samples had four group A haplotypes and accounted for 7.4% of all individuals. These samples were composed of *japonica* and were distributed in Taiwan, Fujian, Anhui, Jiangsu, Shanxi, Beijing, Jilin, Heilongjiang, etc. The average heading date of these samples was 108.9 days. Ten samples had three group A haplotypes and accounted for 6.8% of all individuals. These samples were composed primarily of *japonica* and wild rice, and they were mainly distributed near the Yangtze River in China (Hunan and Jiangxi), as well as in Japan, Indonesia, and high latitudes of the Philippines. The average heading date of these samples was 119.07 days. Twelve samples had two group A haplotypes and accounted for 8.1% of all individuals. They comprised three *japonica*, six *indica* and three wild rice. These individuals were distributed in Hunan and Jiangxi, China, as well as in Indonesia, India, Bangladesh, Bhutan, etc. The average heading date of these samples was 116.39 days. Eighteen samples had one group A haplotype and accounted for 12.2% of all individuals. They comprised three *japonica*, eight *indica*, and three wild rice, and they were distributed in the Philippines, India, Indonesia, Guangdong, Hainan, Bangladesh, and Iran, as well as in Jiangxi and Anhui, China. The average heading date of these samples was 96.3 days. Seventy-six samples had all five group B haplotypes and accounted for 51.4% of all individuals. They were composed of 22 *indica* and 53 wild rice, and they were mainly distributed in India, South and Southeast Asia, and a few regions in southern China. The average heading date of these samples was 171.94 days. These results indicate that if any of the six genes has a group A haplotype, the sample can grow north of the Tropic of Cancer. As cropping regions move northward, a greater number of haplotypes fall into group A (Gao et al., 2014).

## Discussion

Flowering time is one of the best-studied ecologically signiﬁcant traits for adaptation of plants to speciﬁc natural environments. *Hd3a* and *RTF1* are two major florigen genes in the photoperiod regulation pathways. The upstream regulation pathway of *Hd3a* and *RTF1* mainly include long-day suppression pathway, and long-day and short-day promote pathway. Among of them, the long-day suppression genes inhibits the expression of *Hd3a* and *RTF1* under long-day condition, then inhibits the flowering of rice cultivars. So functional change of the long-day suppression genes is the genetic basis of rice flowering in advance. Some studies have also found that the long-day suppression gene as a “bridge” to affect the heading date of cultivated rice (Nemoto et al., 2016). Gao et al. (2014) found that rice flowering is controlled to combinations of *DTH7*, *Ghd7*, or *DTH8* under various environments, especially under long-day conditions. In details, when the three genes are all non-function or weak-function, the heading date of the cultivated rice is obviously earlier than the only one or two genes which are non-function. Zheng et al. (2016) also confirmed that the non-function or weak-function of the long-day suppression genes makes the optimal heading date of the cultivated rice by exploring long-day suppression genes of *Hd1*, *DTH8*, *Ghd7*, and *DTH7* (Zheng et al., 2016). In this study, we not only analyzed the genetic diversity, but also the genetic relationships of these haplotypes, and the relationship between haplotype combination and geographical distribution. These results give us some clues of interaction effects on flowering time difference and skewed distribution in the rice cultivars.

Rice cultivation covers a wide area, including China, South Asia, Southeast Asia, Japan, and South Korea. Among these regions, rice cultivation is most widely distributed in China, which has six rice cropping areas and sixteen sub-regions, including double-crop paddy areas in South China and Central China, single- and double-crop paddy areas in the Southwest Plateau, single-crop paddy areas in North China, early-maturing single-crop paddy areas in Northeast China, and single-crop paddy areas in the dry regions of Northwest China (**Figure S5**). Among of them, There are significant differences in heading date among these regions, with a maximum difference of over 50 days (Wei et al., 2012). Rice is originally a tropical short-day crop; it requires various mutations and haplotype combinations to adapt to the natural environment when expanding into higher latitude cropping areas.

Genetic variation analysis of the long-day suppressor genes *Hd1*, *DTH8*, *DTH7*, *OsCOL4*, *Ghd7*, and *PhyB* indicated that *Hd1* had the largest amount of the genetic variation. However, samples with the group A *Hd1* haplotype were distributed across nearly the entire rice cultivation region from 23°S to 45°N, including South and Southeast Asia, all six rice cropping areas in China, and rice cropping areas in Japan and South Korea (**Figure 2F**). By contrast, the frequency of group A haplotypes of the remaining five long-day suppressor genes gradually increased when moving northward. Compared with *Hd1*, the geographic distribution map of *DTH8* haplotype showed that group A haplotypes mainly appeared in six rice cropping areas in China, Japan and South Korea (**Figure 2E**). Compared with the aforementioned two genes, the haplotype geographic distribution map showed an even narrower latitude range for group A haplotypes of *DTH7* and *OsCOL4*. These were mainly distributed in the double-crop paddy areas in Central China, the single- and double-crop paddy areas in the Southwest Plateau, the single-crop paddy areas in North China, the early-maturing single-crop paddy rice areas in Northeast China, the single-crop paddy areas in the dry regions of Northwest China, and other cropping areas in Japan and Korea (**Figures 2A, B**). *Ghd7* and *PhyB* group A haplotypes were distributed in the single-crop paddy areas in North China, the early-maturing single-crop paddy rice areas in Northeast China, the single-crop paddy areas in the dry regions of Northwest China, and other cropping areas in Japan and Korea (**Figures 2C, D**). In summary, we believe that there is a gradual process of selection acting on long-day suppressor genes as rice cropping areas expand northward. In addition, the numbers of group A haplotypes differ among rice cropping areas, and the number of group A haplotypes of long-day suppressor genes can be increased to further shorten the heading date of cultivated rice in different areas.

Group A haplotypes of *DTH8* were first found in double-crop paddy areas in South China, implying that the group A haplotypes of this gene were selected by nature/human and then accumulated in this area in order to grow cultivated rice that met the heading date requirements of the region (**Figure 2E**). However, with continued northward expansion of cultivated rice, a single *DTH8* gene did not permit cultivated rice to adapt to the heading dates of the double-crop paddy areas in Central China and the single- and double-crop paddy areas in the Southwest Plateau. The haplotypes from *OsCOL4* and *DTH7* group A were selected and then accumulated in these two rice cropping areas (**Figures 2A, B**), gradually permitting development of cultivated rice adapted to the heading date requirements of these regions. It is worth mentioning that in the aforementioned three rice cropping areas, both group A and group B haplotypes were found in *DTH8*, *OsCOL4*, and *DTH7* genes. However, when the cultivated rice cropping area moved northward above 30°N, group A haplotypes completely replaced group B haplotypes of these genes. In the rice-cropping areas north of 30°N, including the sub-regions of double-crop paddy areas in Central China, the single- and double-crop paddy areas in the lower-middle reaches of the Yangtze River, the double-maturing single-crop paddy rice areas in the Sichuan-Shaanxi Basin, the single-crop paddy areas in dry regions of Northwest China, the single-crop paddy areas in North China, the early-maturing single-crop paddy rice areas in Northeast China, and other cropping areas in Japan and Korea, group A haplotypes of *Ghd7* and *PhyB* began to appear and replaced group B haplotypes completely (**Figures 2C, D**). More than four genes among *Hd1*, *DTH8*, *DTH7*, *OsCOL4*, *Ghd7*, and *PhyB* must be of group A in order to meet the heading date requirements of cultivated rice in rice cropping areas north of 30°N.

In conclusion, our study demonstrates that during the northward expansion of cultivated rice, group A haplotypes of the long-day suppressor genes *DTH8*, *DTH7*, *OsCOL4*, *Ghd7*, and *PhyB* were selected to permit adaptation to various latitudes and climate conditions, resulting in the development of cultivated rice varieties with various heading dates in different cropping areas due to the additive effects of multiple genes. In the rice cropping areas north of 30°N, group A haplotypes in long-day suppressor genes replaced group B haplotypes, forming a clear dividing line at 30°N between haplotypes in groups A and B.

## Data Availability Statement

The datasets generated for this study can be found in the NCBI, Genbank number: DTH8 - MT500364 - MT500499; Ghd7 - MT453138 - MT453268; PhyB - MT460250 - MT460374; DTH7 - MT500239 - MT500363; OsCol4 - MT435799 - MT435928; Hd1 - MT500123 - MT500238; Hd6 - MT453303 - MT453434; and Se5 - MT435659 - MT435798.

## Author Contributions

QY and XZ were responsible for study design. LF and SL performed the experiments. JL and YS helped in preparing figures and tables. ZZ, WQ, YLC, and LZ were involved in resources collection. YXC, JW, and LF analyzed the data and wrote the manuscript with editing help from all co-authors. QY and XZ supervised.

## Funding

This work was supported by grants from the National Natural Science Foundation of China (31970237, 31670211) and the Scientific and Technological Innovation Project of the Chinese Academy of Agricultural Sciences.

## Conflict of Interest

The authors declare that the research was conducted in the absence of any commercial or financial relationships that could be construed as a potential conflict of interest.
