# Supplementary figures and images for: A Combination of Long-Day Suppressor Genes Contributes to the Northward Expansion of Rice
Source: Front Plant Sci. 2020 Jun 16;11:864. doi: 10.3389/fpls.2020.00864 (PMC7308711; doi:10.3389/fpls.2020.00864)

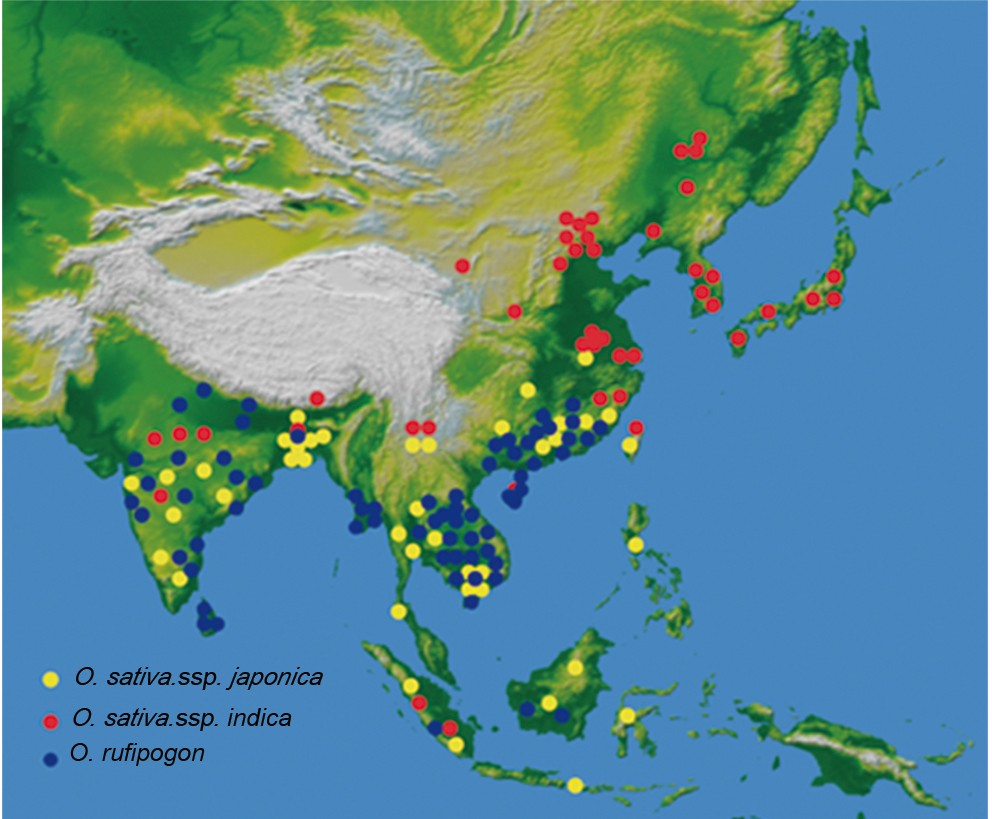

Supplement: Supplementary file 1 [file Image_1.jpg]

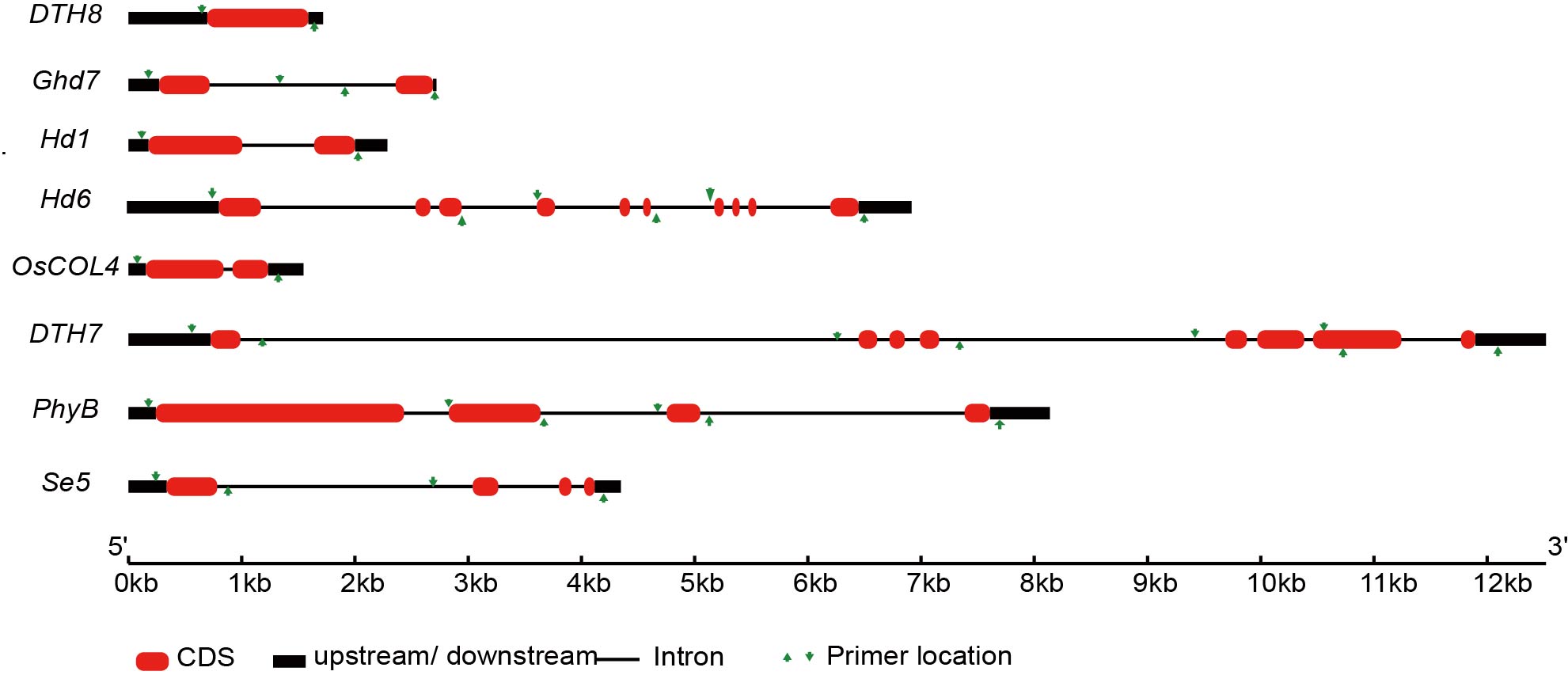

Supplement: Supplementary file 2 [file Image_2.jpg]

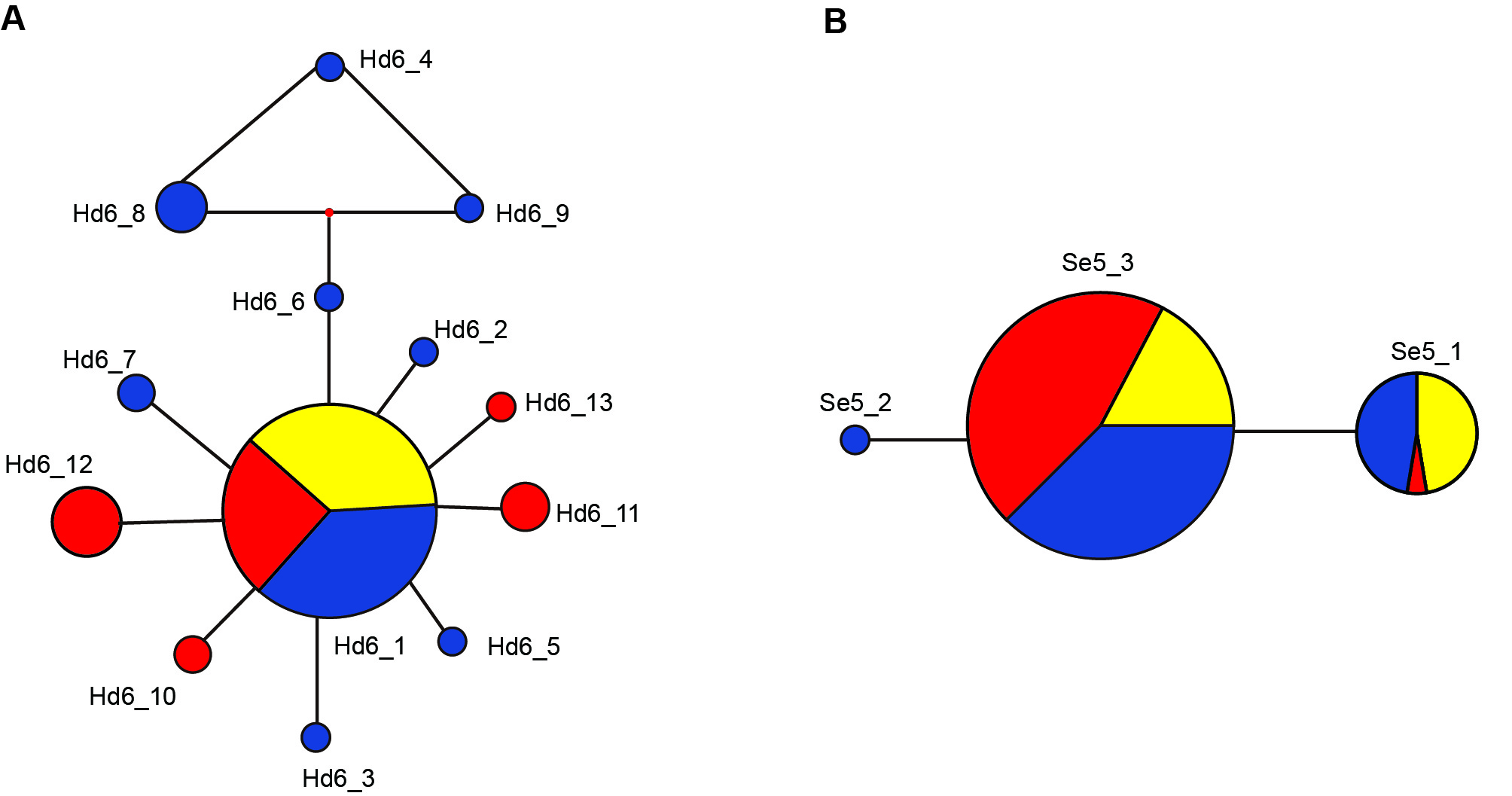

Supplement: Supplementary file 4 [file Image_4.jpg]

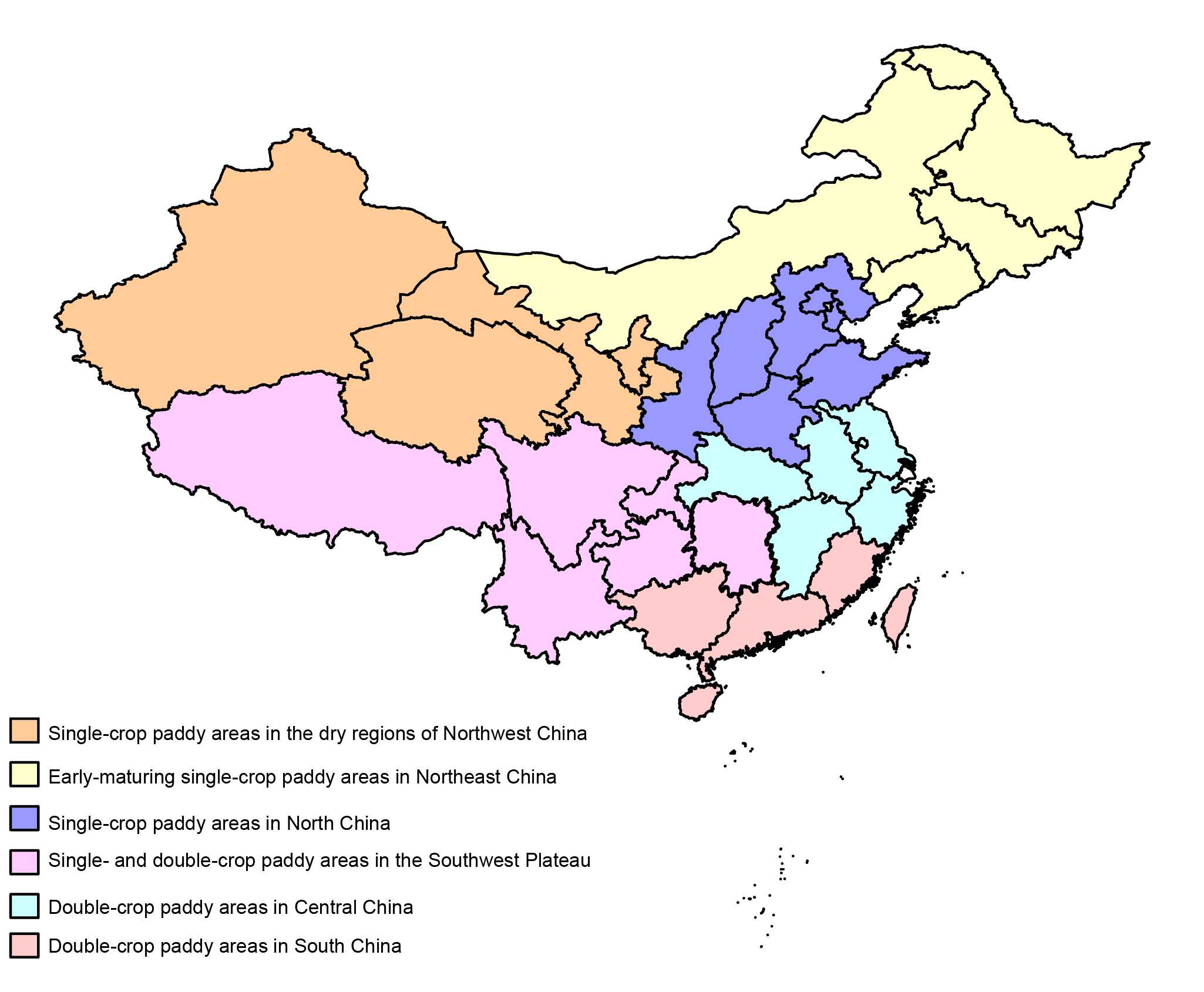

Supplement: Supplementary file 5 [file Image_5.jpg]
