# Supplementary material for: A Combination of Long-Day Suppressor Genes Contributes to the Northward Expansion of Rice
Source: Front Plant Sci. 2020 Jun 16;11:864. doi: 10.3389/fpls.2020.00864 (PMC7308711; doi:10.3389/fpls.2020.00864)
Supplement: Supplementary file 3 [file Image_3.pdf]

A

DTH8

894 bp

100 bp

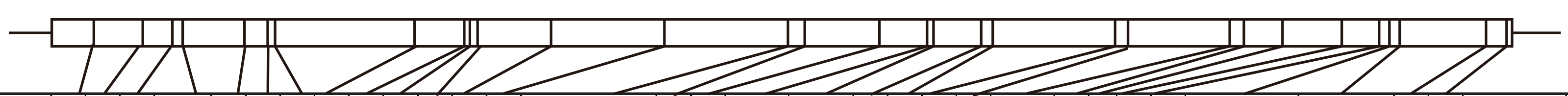

Position	26	56	74	80	118	132	139	222	256	261	282	306	375	451	461	507	536	539	569	576	651	659	712	730	754	790	813	819	825	878	893	No.of cvs			f / nf	Group
Position variation	H9L	L19S	E25A	Stop	S40G	-	G47S	-	S86A	-	-	-	-	Stop	D154A	E169D	-	-	Y190S	-	-	D220V	indel	A244T	G252S	I264V	-	indel	indel	Stop	N298S	I	J	W		
DTH8_1	A	T	A	a	A	C	G	T	T	G	G	A	C	GAGAAGGC	A	G	GCG	3bp	A	C	C	A	GCG	G	G	A	A	6bp	GCGG4	TT	A	0	1	1	f	groupA
DTH8_2	A	T	A	a	A	C	G	G	T	G	G	A	C	8bp	A	G	GCG	3bp	A	C	C	A	GCG	G	G	A	A	6bp	GCGG4	TT	A	4	0	0	nf	groupA
DTH8_3	A	T	A	a	A	C	G	G	T	G	G	A	C	GAGAAGGC	A	G	GCG	GCG	A	C	C	A	GCG	G	G	A	A	6bp	GCGG4	TT	A	0	0	10	f	groupA
DTH8_4	A	T	A	a	A	C	G	G	T	G	G	A	C	GAGAAGGC	A	G	GCG	3bp	A	C	C	A	GCG	G	G	A	A	6bp	GCGG4	TT	A	0	34	1	f	groupA
DTH8_5	A	T	A	19bp	A	C	G	G	T	G	G	A	C	GAGAAGGC	A	G	GCG	3bp	A	C	C	A	GCG	G	G	A	A	6bp	GCGG4	TT	A	0	1	0	nf	groupA
DTH8_6	T	C	C	a	A	T	G	G	G	G	G	C	C	GAGAAGGC	C	G	3bp	3bp	C	C	C	C	GCG	G	G	A	A	AGGCGG	GCGG4	GA	G	0	0	7	f	groupB
DTH8_7	A	C	C	a	A	C	G	G	G	G	A	C	A	GAGAAGGC	C	T	GCG	3bp	C	C	C	C	GCG	G	G	A	C	6bp	12bp	GA	G	0	0	1	nf	groupB
DTH8_8	A	C	C	a	A	C	G	G	G	G	G	C	C	GAGAAGGC	C	T	GCG	3bp	C	C	C	C	GCG	G	G	A	C	6bp	12bp	GA	G	0	0	1	nf	groupB
DTH8_9	A	C	C	a	A	C	G	G	G	G	G	C	C	GAGAAGGC	C	G	3bp	3bp	C	A	C	C	GCG	A	G	G	C	6bp	12bp	GA	G	0	0	2	nf	groupB
DTH8_10	A	C	C	a	A	C	A	G	G	A	G	C	C	GAGAAGGC	C	G	3bp	3bp	C	A	C	C	GCG	G	G	A	C	6bp	12bp	GA	G	0	0	1	nf	groupB
DTH8_11	A	C	C	a	A	C	G	G	G	G	G	C	C	GAGAAGGC	C	G	3bp	3bp	C	A	C	C	GCG	G	G	A	C	6bp	12bp	GA	G	0	0	1	nf	groupB
DTH8_12	A	C	C	a	G	C	G	G	G	G	G	C	C	GAGAAGGC	C	G	3bp	3bp	C	C	C	C	3bp	G	G	A	A	6bp	GCGG3	TT	G	0	0	3	f	groupB
DTH8_13	A	C	C	a	A	C	G	G	G	G	G	C	C	GAGAAGGC	C	G	GCG	GCG	C	C	T	C	GCG	G	G	A	A	6bp	GCG3	TT	G	1	0	0	f	groupB
DTH8_14	A	C	C	a	A	C	G	G	G	G	G	C	C	GAGAAGGC	C	G	GCG	GCG	C	C	T	C	GCG	G	G	A	A	6bp	GCGG4	TT	G	7	8	5	f	groupB
DTH8_15	A	C	C	a	A	C	G	G	G	G	G	C	C	GAGAAGGC	C	G	GCG	GCG	C	C	T	C	GCG	G	G	A	A	6bp	GCG3	TT	G	18	3	11	f	groupB
DTH8_16	A	C	C	a	A	C	G	G	G	G	G	C	C	GAGAAGGC	C	G	3bp	3bp	C	C	T	C	GCG	G	G	A	A	6bp	GCGG4	TT	G	3	0	1	f	groupB
DTH8_17	A	C	C	a	A	C	G	G	G	G	G	C	C	GAGAAGGC	C	G	3bp	3bp	C	C	C	C	GCG	G	A	A	A	6bp	GCGG4	TT	G	0	0	8	f	groupB
DTH8_18	A	C	C	a	A	C	G	G	G	G	G	C	C	GAGAAGGC	C	G	3bp	3bp	C	C	C	C	GCG	G	G	A	A	6bp	GCGG4	TT	G	0	0	3	f	groupB

B

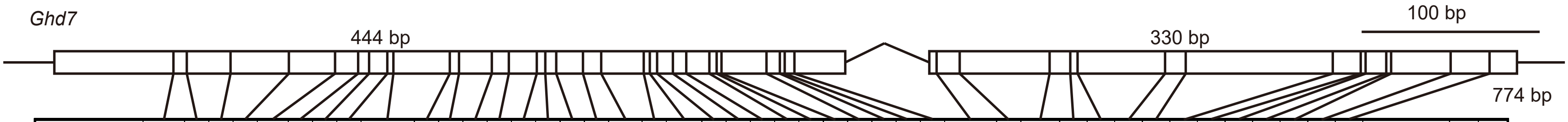

Position	67	74	99	132	157	171	177	187	190	223	227	246	255	271	276	282	297	307	331	335	339	348	355	368	373	375	400	407	409	416	448	461	511	523	527	577	588	671	686	689	701	703	737	759	No.of cvs			f/nf	Group
Position Variation	C23S	H25R	-	-	Stop	-	N59K	G63S	indel	D75N	D75G	-	-	H91D	-	-	-	L103P	G111S	A112G	-	-	V119I	G123E	P125A	P126A	R134G	G136V	G137S	Q139R	indel	C154Y	A171G	D175N	V176D	E139K	-	R225T	G228D	R230L	E234V	P235A	Y246S	-	I	J	W		
Ghd7_35	TC	A	C	C	G	T	C	G	3bp	G	A	C	C	C	G	G	C	CC	G	CC	C	G	G	A	C	C	G	G	G	G	3bp	G	GC	G	T	G	G	G	G	G	A	C	A	G	0	0	1	f	groupA
Ghd7_36	TC	A	C	C	G	T	C	G	3bp	G	A	C	C	C	G	G	C	CC	G	CC	C	G	G	A	C	C	G	G	G	G	3bp	G	GC	G	A	A	G	G	G	G	A	C	A	G	0	0	3	f	groupA
Ghd7_38	TC	A	C	C	G	C	C	G	3bp	G	A	C	C	C	G	G	C	CC	G	CC	C	G	G	A	C	C	G	G	G	G	3bp	G	GC	G	A	G	G	G	G	T	A	C	A	G	0	1	0	f	groupA
Ghd7_39	TC	A	C	C	G	C	C	G	3bp	G	A	C	C	C	G	G	C	CC	G	CC	C	G	G	A	C	C	G	G	G	G	3bp	G	GC	G	A	G	G	G	A	G	A	C	A	G	0	1	0	f	groupA
Ghd7_40	TC	A	C	C	T	C	C	G	3bp	G	A	C	C	C	G	G	C	CC	G	CC	C	G	G	A	C	C	G	G	G	G	3bp	G	GC	G	A	G	G	G	G	G	A	C	A	G	0	2	0	nf	groupA
Ghd7_41	TC	A	C	C	G	C	C	G	3bp	G	A	C	C	C	G	G	C	CC	G	CC	C	G	G	A	C	C	G	G	G	G	3bp	G	GC	G	A	G	G	G	G	G	A	C	A	G	9	31	0	f	groupA
Ghd7_1	TG	A	C	T	G	C	C	G	CGA	G	A	C	C	C	G	A	C	CT	G	CC	C	G	G	G	C	C	C	G	G	A	3bp	G	GC	G	T	G	G	G	G	G	A	C	A	G	1	0	3	f	groupB
Ghd7_2	TG	A	C	T	G	C	C	G	CGA	G	A	C	C	C	G	G	C	TT	G	CC	C	G	G	G	C	C	C	G	G	G	3bp	G	GC	G	T	G	G	G	G	G	A	C	A	G	0	0	1	f	groupB
Ghd7_3	TC	A	C	C	G	C	C	G	3bp	G	A	C	C	C	G	G	C	CC	G	GC	A	G	G	G	C	C	G	G	A	G	3bp	G	GC	A	T	G	G	G	G	G	A	C	A	G	1	1	0	f	groupB
Ghd7_4	TC	A	C	C	G	C	C	A	3bp	A	A	C	C	C	G	G	T	CC	G	CC	C	G	G	G	C	C	G	T	G	G	3bp	A	GC	G	T	G	G	G	G	G	A	C	A	G	0	0	1	f	groupB
Ghd7_5	TC	A	C	C	G	C	C	A	3bp	A	A	C	C	C	G	G	T	CC	G	CC	C	G	G	G	C	C	G	G	G	G	3bp	G	GC	G	T	G	G	G	G	G	A	C	A	A	0	0	2	f	groupB
Ghd7_6	TC	A	C	C	G	C	G	A	3bp	A	A	C	C	C	G	G	T	CC	G	CC	C	G	G	G	C	C	G	G	G	G	GTG	G	GC	G	T	G	G	G	G	G	A	C	A	G	0	0	1	f	groupB
Ghd7_7	TC	A	C	C	G	C	C	A	3bp	A	A	C	C	C	G	G	T	CC	G	CC	C	G	G	G	C	C	G	G	G	G	3bp	G	GC	G	T	G	G	G	G	G	A	C	A	G	0	0	3	f	groupB
Ghd7_8	TC	A	A	C	G	C	C	G	3bp	G	A	C	C	C	G	G	C	CC	G	CC	C	G	G	G	C	C	G	G	G	G	3bp	G	GG	G	T	G	G	G	G	G	A	C	A	G	0	0	2	f	groupB
Ghd7_9	TC	A	C	C	G	C	C	G	3bp	G	A	G	C	C	G	G	C	CC	G	CC	C	G	G	G	C	C	G	G	A	G	3bp	G	CC	G	T	G	G	G	G	G	A	C	A	G	1	0	5	f	groupB
Ghd7_10	TC	A	C	C	G	C	C	G	3bp	G	A	C	C	C	G	G	C	CC	G	CC	C	G	G	G	C	T	G	G	A	G	3bp	G	GC	G	T	G	T	G	G	G	T	C	A	G	0	0	1	f	groupB
Ghd7_11	TC	A	C	C	G	C	C	G	3bp	G	A	C	C	C	G	G	C	CC	A	CC	C	G	A	G	C	C	G	G	G	G	3bp	G	GC	G	T	G	G	G	G	G	A	C	A	G	0	3	0	f	groupB
Ghd7_12	CC	A	C	C	G	C	C	G	3bp	G	A	C	C	C	G	G	C	CC	G	CC	C	G	A	G	C	C	G	G	G	G	3bp	G	GC	G	T	G	G	G	G	G	A	C	A	G	3	0	0	f	groupB
Ghd7_13	TC	A	C	C	G	C	C	G	3bp	G	A	C	C	C	G	G	C	CC	G	CC	C	G	A	G	C	C	G	G	G	G	3bp	G	GC	G	T	G	G	G	G	G	A	C	A	G	0	0	2	f	groupB
Ghd7_14	TC	G	C	C	G	C	C	G	3bp	G	A	C	C	C	G	G	C	CC	G	CC	C	T	A	G	C	C	G	G	G	G	3bp	G	GC	G	T	G	G	G	G	G	A	C	A	G	0	0	1	f	groupB
Ghd7_15	TC	G	C	C	G	C	C	G	3bp	G	A	C	C	C	G	G	C	CC	G	CC	C	G	A	G	C	C	G	G	G	G	3bp	G	GC	G	T	G	G	G	G	G	A	C	C	G	0	0	1	f	groupB
Ghd7_16	TC	G	C	C	G	C	C	G	3bp	G	A	C	C	C	G	G	C	CC	G	CC	C	G	A	G	C	C	G	G	G	G	3bp	G	GC	G	T	G	G	G	G	G	A	C	A	G	0	0	2	f	groupB
Ghd7_17	TC	A	C	C	G	C	C	G	3bp	G	A	C	C	C	G	G	C	CC	G	CC	C	T	G	G	C	C	G	G	G	G	3bp	G	GC	G	T	G	G	G	G	G	A	C	A	G	0	0	1	f	groupB
Ghd7_18	TC	A	C	C	G	C	C	G	3bp	G	A	C	C	C	G	G	C	CC	G	CC	C	T	A	G	C	C	G	G	G	G	3bp	G	GG	G	T	G	G	G	G	G	A	C	A	G	0	0	1	f	groupB
Ghd7_19	TC	A	C	C	G	C	C	G	3bp	G	A	C	C	C	G	G	C	CC	G	CC	C	T	A	G	C	C	G	G	G	G	3bp	G	GC	G	T	G	G	G	G	G	A	C	A	G	0	0	1	f	groupB
Ghd7_20	TC	A	C	C	G	C	C	G	3bp	G	A	C	C	C	G	G	C	CC	G	CC	C	G	G	G	C	C	G	G	A	G	3bp	G	GC	G	T	G	G	C	G	G	A	G	A	G	2	0	0	f	groupB
Ghd7_21	TC	A	C	C	G	C	C	G	3bp	G	A	C	C	C	G	G	C	CC	G	CC	C	G	G	G	C	C	G	G	A	G	3bp	G	GC	G	T	A	G	G	G	G	A	G	A	G	1	0	0	f	groupB
Ghd7_22	TC	A	C	C	G	C	C	G	3bp	G	A	C	C	C	G	G	C	CC	G	CC	C	G	G	G	G	C	G	G	A	G	3bp	G	GC	G	T	G	G	G	G	G	A	G	A	G	1	0	0	f	groupB
Ghd7_23	TC	A	C	C	G	C	C	G	3bp	G	A	C	C	C	G	G	C	CC	G	CC	C	G	G	G	C	C	G	G	A	G	3bp	G	GC	G	T	G	G	G	G	G	A	G	A	G	15	3	2	f	groupB
Ghd7_24	TC	A	C	C	G	C	C	G	3bp	G	A	C	C	C	G	G	C	CC	G	CC	C	G	G	G	C	C	G	G	G	G	3bp	G	GC	G	T	A	G	G	G	G	A	C	A	G	0	0	3	f	groupB
Ghd7_25	TC	A	C	C	G	C	C	G	3bp	G	A	C	T	C	G	G	C	CC	G	CC	C	G	G	G	C	C	G	G	G	G	3bp	G	GC	G	A	A	G	G	G	G	A	C	A	G	0	0	2	f	groupB
Ghd7_26	TC	A	C	C	G	C	C	G	3bp	G	A	C	C	C	G	G	C	CC	G	CC	C	G	G	G	C	C	G	G	G	G	3bp	G	GC	G	A	A	G	G	G	G	A	C	A	G	0	0	1	f	groupB
Ghd7_27	TC	A	C	C	G	C	C	G	3bp	G	A	C	C	G	G	G	C	CC	G	CC	C	G	G	G	C	C	G	G	G	G	3bp	G	GC	G	A	G	G	G	G	G	A	C	A	G	0	0	1	f	groupB
Ghd7_28	TC	A	C	C	G	C	C	G	3bp	G	A	C	C	G	G	G	C	CC	G	CC	C	G	G	G	C	C	G	G	G	G	3bp	G	GC	G	A	A	G	G	G	G	A	C	A	G	0	0	1	f	groupB
Ghd7_29	TC	A	C	C	G	C	C	G	3bp	G	A	C	C	C	G	G	C	CC	G	CC	C	G	G	G	C	C	G	G	A	G	3bp	G	GC	G	T	G	G	G	G	G	A	C	A	G	0	2	2	f	groupB
Ghd7_30	TC	A	C	C	G	C	C	G	3bp	G	G	C	C	C	G	G	C	CC	G	CC	C	G	G	G	C	C	G	G	A	G	3bp	G	GC	G	T	G	G	G	G	G	T	C	A	G	0	0	1	f	groupB
Ghd7_31	TC	A	C	C	G	C	C	G	3bp	G	A	C	C	C	G	G	C	CC	G	CC	C	G	G	G	C	C	G	G	A	G	3bp	G	GC	G	T	G	G	G	G	G	T	C	A	G	0	0	2	f	groupB
Ghd7_32	TC	A	C	C	G	C	C	G	3bp	G	A	C	C	C	C	G	C	CC	G	CC	C	G	G	G	C	C	G	G	G	G	3bp	G	GC	G	T	G	G	G	G	G	A	C	A	G	0	0	1	f	groupB
Ghd7_33	TC	A	C	C	G	C	C	G	3bp	A	A	C	C	C	G	G	C	CC	G	CC	C	G	G	G	C	C	G	G	G	G	3bp	G	GC	G	T	G	G	G	G	G	A	C	A	G	0	0	1	f	groupB
Ghd7_34	TC	A	C	C	G	C	C	G	3bp	G	A	C	C	C	G	G	C	CC	G	CC	C	G	G	G	C	C	G	G	G	G	3bp	G	GC	G	T	G	G	G	G	G	A	C	A	G	0	0	3	f	groupB
Ghd7_37	TC	A	C	C	G	C	C	G	3bp	G	A	C	C	C	G	G	C	CC	G	CA	C	G	G	G	C	C	G	G	G	G	3bp	G	GC	G	A	G	G	G	G	G	A	C	A	G	0	0	1	f	groupB

C *Hd1*

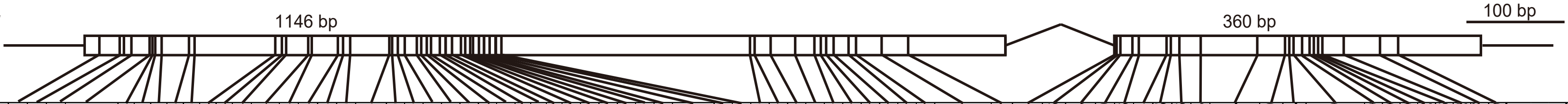

Position	18	43	49	58	81	82	87	95	134	137	241	245	251	278	283	319	320	331	379	382	390	402	438	439	442	443	469	472	478	484	487	490	493	496
Position Variation	-	G15R	G17R	indel	-	S28G	-	W32L	V45A	V46A	A81P	C82Y	O84R	A93V	A95S	H107Y	H107P	indel	I127V	T128I	-	-	-	G147S	G148N	G148N	I157L	I158L	S160T	D162N	S163P	D164N	N165D	N166D
Hd1_26	T	G	G	AGG1	G	A	C	G	T	T	G	G	GC	C	G	T	A	m	A	A	G	C	C	G	G	A	C	C	T	G	T	G	A	A
Hd1_27	T	G	G	AGG1	G	A	C	G	T	T	G	G	GC	C	G	T	A	m	A	A	G	C	C	G	G	A	C	C	T	G	T	G	A	A
Hd1_28	T	G	G	AGG1	G	A	C	G	T	T	G	G	GC	C	G	C	A	m	A	A	G	C	C	G	G	A	C	C	T	G	T	G	A	A
Hd1_29	T	G	G	AGG1	G	A	C	G	T	T	G	G	GC	C	G	C	A	36bp	A	A	G	C	C	G	G	A	C	C	T	G	T	G	A	A
Hd1_30	T	G	G	AGG1	G	A	C	G	T	T	G	G	GC	C	G	C	A	m	A	A	G	C	C	G	G	A	C	C	T	G	T	G	A	A
Hd1_31	T	G	G	AGG1	G	A	C	G	T	T	G	G	GC	C	G	C	A	m	A	A	G	C	C	G	G	A	C	C	T	G	T	G	A	A
Hd1_32	T	G	G	AGG1	G	A	C	G	T	T	G	G	GC	C	G	C	A	m	A	A	G	C	C	G	G	A	C	C	T	G	T	G	A	A
Hd1_33	T	G	G	AGG1	G	A	C	G	T	T	G	G	GC	C	G	C	A	m	A	A	G	C	C	G	G	A	C	C	T	G	T	G	A	A
Hd1_34	T	G	G	AGG1	G	A	C	G	T	T	G	G	GC	C	G	C	A	m	A	A	G	C	C	G	G	A	C	C	T	G	T	G	A	A
Hd1_39	T	G	G	AGG1	G	A	C	G	T	T	G	G	GC	C	G	C	A	m	A	A	G	C	C	G	G	A	C	C	T	G	T	G	A	A
Hd1_1	T	G	G	AGG1	G	A	C	G	T	T	G	G	AA	C	G	C	A	m	A	A	G	C	C	G	G	G	A	A	T	G	T	A	A	A
Hd1_2	T	G	G	AGG1	G	A	C	G	T	T	G	A	AA	C	G	C	A	m	A	A	G	C	C	G	G	G	A	A	T	G	T	A	A	A
Hd1_3	T	G	G	6bp	G	A	C	G	T	T	G	G	AA	C	G	C	A	m	A	A	G	C	C	G	G	G	A	A	T	G	T	A	A	A
Hd1_4	T	G	G	AGG1	G	A	C	G	T	T	G	G	AA	C	G	C	A	m	A	A	G	C	C	G	G	G	A	A	T	G	T	A	A	A
Hd1_5	T	G	G	AGG1	G	A	C	G	T	T	G	G	AA	C	G	C	A	m	A	A	G	C	C	G	G	G	A	A	T	G	T	A	A	A
Hd1_6	T	G	G	AGG1	G	A	C	G	T	G	G	G	AA	C	G	C	A	m	A	A	G	C	C	G	G	G	A	A	T	G	T	A	A	A
Hd1_7	T	G	G	AGG1	G	A	C	G	T	T	G	G	AA	C	G	C	A	m	A	A	G	C	C	G	G	G	A	A	T	G	T	A	A	A
Hd1_8	T	G	G	AGG1	G	A	C	G	T	T	G	G	AA	C	G	C	A	m	A	A	G	C	C	G	G	G	A	A	T	G	T	A	A	A
Hd1_9	T	G	G	AGG1	G	A	C	G	T	T	G	G	AA	C	G	C	A	m	A	A	G	C	C	G	G	G	A	A	T	G	T	A	A	A
Hd1_10	T	G	G	AGG1	G	A	C	G	T	T	G	G	AA	C	G	C	A	m	A	A	G	C	C	G	G	G	A	A	T	G	T	A	A	A
Hd1_11	T	G	G	AGG1	G	A	C	G	T	T	G	G	AA	C	G	C	A	m	A	A	G	C	C	G	G	G	A	A	T	G	T	A	A	A
Hd1_12	T	G	G	AGG1	G	A	C	G	T	T	G	G	AA	C	G	C	A	m	A	A	G	C	C	G	G	G	A	A	T	G	T	A	A	A
Hd1_13	T	G	G	AGG1	G	A	C	T	T	T	G	G	GC	C	G	C	A	m	A	A	A	C	C	G	G	A	C	C	T	G	T	A	A	A
Hd1_14	T	G	G	AGG1	G	A	C	T	T	T	G	G	GC	C	G	C	A	m	A	A	A	C	C	G	A	A	C	C	T	G	T	A	A	A
Hd1_15	T	G	G	6bp	G	A	C	G	T	T	G	G	GC	T	G	C	C	m	A	A	G	C	C	A	G	A	C	C	T	G	T	A	A	A
Hd1_16	T	G	C	AGG1	G	A	C	G	T	T	C	G	GC	C	G	C	A	m	A	A	G	C	C	G	G	A	C	C	T	G	T	A	A	A
Hd1_17	T	G	C	AGG1	G	A	C	G	T	T	G	G	GC	C	G	C	A	m	A	A	G	C	C	G	G	A	C	C	T	G	T	A	A	A
Hd1_18	T	G	C	AGG1	G	A	C	G	T	T	G	G	GC	C	G	C	A	m	A	A	G	C	C	G	G	A	C	C	T	G	T	A	A	A
Hd1_19	T	G	G	AGG1	G	A	C	G	T	T	G	G	GC	C	T	C	A	m	A	A	G	C	C	G	G	A	C	C	T	G	T	G	A	A
Hd1_20	T	A	G	AGG1	G	A	C	G	T	T	G	G	GC	C	G	C	A	m	A	A	G	C	C	G	G	A	C	C	T	G	T	G	G	G
Hd1_21	T	G	G	AGG1	G	A	C	G	C	T	G	G	GC	C	G	C	A	m	A	A	G	C	C	G	G	A	C	C	T	G	T	A	A	A
Hd1_22	T	G	G	AGG1	G	A	C	G	C	T	G	G	GC	C	G	C	A	m	A	A	G	C	C	G	G	A	C	C	T	G	T	G	A	A
Hd1_23	T	G	G	AGG1	G	A	C	G	C	T	G	G	GC	C	G	C	A	m	A	A	G	C	T	G	G	A	C	C	T	G	T	G	A	A
Hd1_24	T	G	G	AGG1	G	A	C	G	C	T	G	G	GC	C	G	C	A	m	A	A	G	C	C	G	G	A	C	C	T	G	T	G	A	A
Hd1_25	T	G	G	AGG1	G	A	C	G	T	T	G	G	GC	C	G	C	A	m	A	A	G	C	C	G	G	A	C	C	T	G	T	G	A	A
Hd1_35	T	G	G	AGG1	G	A	C	G	T	T	G	G	GC	C	G	C	A	m	A	A	G	C	C	G	G	A	C	C	T	G	T	G	G	A
Hd1_36	T	G	G	AGG1	G	A	C	G	T	T	G	G	GC	C	G	C	A	m	A	A	G	C	C	G	G	A	C	C	T	G	T	G	G	A
Hd1_37	T	G	G	AGG1	G	A	C	G	T	T	G	G	GC	C	G	C	A	m	A	A	G	C	C	G	G	A	C	C	T	G	T	G	G	A
Hd1_38	T	G	G	AGG1	G	A	C	G	T	T	G	G	GC	C	G	C	A	m	A	A	G	C	C	G	G	A	C	C	T	G	T	G	A	A
Hd1_40	C	G	G	AGG1	C	G	A	G	T	T	G	G	GC	C	G	C	A	m	G	G	G	G	C	G	G	A	C	C	A	A	C	G	A	A
Hd1_41	T	G	G	AGG1	G	A	C	G	T	T	G	G	GC	C	G	C	A	m	G	G	G	G	C	G	G	A	C	C	A	A	C	G	A	A
Hd1_42	T	G	G	AGG1	C	G	A	G	T	T	G	G	GC	C	G	C	A	m	G	G	G	G	C	G	G	A	C	C	A	A	C	G	A	A

Position	499	504	507	508	827	833	854	885	908	916	923	932	951	961	992	1021	1146	1153	1169	1176	1211	1215	1226	1253	1324	1359	1364	1369	1380	1389	1390	1392	1396	1432	1477	1499	No.of cvs I	J	W	f / nf	Group
Position Variation	indel	-	-	indel	S276N	S278N	F285Y	-	N303S	E306K	N308S	Q311R	-	indel	E331V	Stop	S382R	Stop	-	-	S404F	S405R	P409L	G418V	indel	-	E455A	Stop	-	-	Stop	-	-	Q478E	S493G	Stop					
Hd1_26	AAC	C	T	h	G	G	T	C	A	G	A	G	A	CAG3	A	n	C	TT	C	A	C	A	C	G	AGA	T	A	AAGA	T	A	C	A	C	C	G	G	0	5	0	f	groupA
Hd1_27	AAC	C	T	h	G	G	T	C	A	G	A	G	A	CAG3	A	43bp	C	TT	C	A	C	A	C	G	AGA	T	A	AAGA	T	A	C	A	C	C	G	G	0	6	0	f	
Hd1_28	AAC	C	T	h	G	G	T	C	A	G	A	G	A	CAG3	A	n	C	TT	C	A	C	A	C	G	AGA	T	A	AAGA	T	A	C	A	C	C	A	G	0	2	1	f	
Hd1_29	AAC	C	T	h	G	G	T	C	A	G	A	G	A	CAG3	A	n	C	TT	C	A	C	A	C	G	AGA	T	A	AAGA	T	A	C	A	C	C	G	G	0	2	0	f	
Hd1_30	AAC	C	T	h	G	G	T	C	A	G	A	G	A	CAG3	A	n	C	TT	C	A	C	A	C	G	AGA	T	A	AAGA	T	A	C	A	C	C	G	G	0	3	0	f	
Hd1_31	AAC	C	T	h	G	G	T	C	A	G	A	A	A	CAG3	A	n	C	TT	C	A	C	A	C	G	AGA	T	A	AAGA	T	A	C	A	C	C	G	G	17	0	5	f	
Hd1_32	AAC	C	T	h	G	G	T	C	A	G	A	A	A	CAG3	A	n	C	TT	C	A	C	A	C	G	AGA	T	A	4bp	T	A	C	A	C	C	G	G	4	1	0	nf	
Hd1_33	AAC	C	T	h	G	G	T	C	A	G	A	A	A	CAG3	A	n	C	TT	C	A	C	A	C	G	AGA	T	A	AAGA	T	A	C	A	C	C	G	G	0	0	1	f	
Hd1_34	AAC	C	T	h	G	G	T	C	A	G	A	A	A	CAG3	A	n	C	TT	C	A	C	A	C	G	AGA	T	A	AAGA	T	A	C	A	C	C	G	G	1	0	0	f	
Hd1_39	AAC	C	T	h	G	G	T	C	A	G	A	A	A	CAG3	A	n	C	TT	C	A	C	A	C	G	AGA	T	A	AAGA	T	A	C	A	C	C	G	G	1	0	1	f	
Hd1_1	AAC	T	C	a	G	G	T	C	A	G	A	A	A	CAG3	A	n	C	2bp	C	A	C	C	C	G	AGA	T	A	AAGA	T	A	C	A	C	C	A	G	1	0	0	nf	groupB
Hd1_2	AAC	C	T	b	G	G	T	C	A	G	A	A	A	CAG3	A	n	C	TT	C	A	C	C	C	G	AGA	T	A	AAGA	T	A	C	A	C	C	A	G	0	1	0	f	
Hd1_3	AAC	C	T	c	G	A	T	C	A	G	A	A	A	CAG3	A	n	C	2bp	C	A	C	C	C	G	AGA	T	A	AAGA	T	A	C	A	C	C	A	G	0	1	0	nf	
Hd1_4	AAC	C	T	c	G	G	T	C	A	G	A	A	A	CAG3	A	n	C	2bp	C	A	C	C	C	G	AGA	T	A	AAGA	T	A	C	A	C	C	A	G	0	1	0	nf	
Hd1_5	AAC	C	T	c	G	G	T	C	A	G	A	A	A	CAG3	A	n	C	TT	C	A	C	C	C	G	AGA	T	A	AAGA	T	A	T	A	C	C	A	G	0	1	0	nf	
Hd1_6	AAC	C	T	c	G	G	T	C	A	G	A	A	A	CAG3	A	n	C	TT	C	A	C	C	C	G	AGA	T	A	AAGA	T	A	C	A	C	C	A	G	0	1	0	f	
Hd1_7	AAC	C	T	c	G	G	T	C	A	G	A	A	A	CAG3	A	n	C	TT	C	A	C	C	C	G	3bp	T	A	AAGA	T	A	C	A	C	C	A	G	0	1	0	f	
Hd1_8	AAC	C	T	d	G	G	T	C	A	G	A	A	A	CAG3	A	n	C	TT	C	A	C	C	C	G	AGA	T	A	AAGA	T	A	C	A	C	C	A	G	1	7	0	f	
Hd1_9	AAC	C	T	d	G	G	T	C	A	G	A	A	A	CAG3	A	n	C	TT	C	A	C	C	C	G	3bp	T	A	AAGA	T	A	C	A	C	C	A	G	0	1	0	f	
Hd1_10	AAC	C	T	e	G	G	T	C	A	G	A	A	A	CAG3	A	n	C	TT	C	A	C	C	C	G	AGA	T	A	AAGA	T	A	C	A	C	C	A	G	0	2	0	f	
Hd1_11	AAC	C	T	c	G	G	T	C	A	G	A	A	A	CAG3	A	n	C	2bp	C	A	C	C	C	G	AGA	T	A	AAGA	T	A	C	A	C	C	G	G	1	3	2	nf	
Hd1_12	AAC	C	T	d	G	G	T	C	A	G	A	A	A	CAG3	A	n	C	2bp	C	A	C	C	C	G	AGA	T	A	AAGA	T	A	C	A	C	C	G	G	8	1	0	nf	
Hd1_13	AAC	C	T	f	G	G	T	C	A	G	A	A	A	CAG3	T	n	C	TT	C	A	C	C	T	G	AGA	T	A	AAGA	T	A	C	A	C	C	G	A	0	0	1	nf	
Hd1_14	3bp	C	C	g	G	G	T	C	A	G	A	A	A	CAG3	T	n	C	TT	C	A	C	C	T	G	AGA	T	A	AAGA	T	A	C	A	C	C	G	G	0	0	1	f	
Hd1_15	GAC	C	T	h	G	G	T	C	A	G	A	A	A	CAG3	A	n	C	TT	C	A	C	C	C	T	AGA	T	A	AAGA	T	A	C	A	C	C	G	G	0	0	2	f	
Hd1_16	AAC	C	T	i	G	G	T	C	A	G	A	A	A	CAG3	A	n	G	TT	C	A	C	C	C	G	AGA	T	A	AAGA	T	A	C	A	C	C	G	G	0	0	1	f	
Hd1_17	AAC	C	T	i	G	G	T	C	A	G	A	A	A	CAG3	A	n	G	TT	C	A	C	C	C	G	AGA	T	A	AAGA	T	A	C	A	C	C	G	G	1	0	7	f	
Hd1_18	AAC	C	T	h	G	G	T	C	A	G	A	A	A	CAG3	A	n	G	TT	C	A	C	C	C	G	AGA	T	A	AAGA	T	A	C	A	C	C	G	G	0	0	1	f	
Hd1_19	AAC	C	T	h	G	G	T	T	A	G	A	A	A	CAG3	A	n	C	TT	C	A	C	C	C	G	AGA	T	A	AAGA	T	A	C	A	C	C	G	G	0	0	1	f	
Hd1_20	AAC	C	C	i	G	G	T	C	A	G	A	A	A	CAG3	A	n	C	TT	C	T	T	C	C	G	AGA	T	A	AAGA	T	A	C	A	C	C	G	G	0	0	2	f	
Hd1_21	AAC	T	T	i	G	G	T	C	A	G	A	A	A	CAG3	A	n	C	TT	C	A	C	C	C	G	AGA	T	A	AAGA	T	A	C	A	C	C	G	G	0	0	2	f	
Hd1_22	AAC	C	T	h	G	G	T	C	A	G	A	A	A	CAG3	A	n	C	TT	C	A	C	C	C	G	AGA	T	A	AAGA	T	A	C	A	C	C	G	G	0	0	1	f	
Hd1_23	AAC	C	T	h	G	G	T	C	A	G	A	A	A	CAG3	A	n	C	TT	C	A	C	C	T	G	AGA	T	A	AAGA	T	A	C	A	C	C	G	G	0	0	1	f	
Hd1_24	AAC	C	T	h	G	G	T	C	A	G	A	A	A	CAG3	A	n	C	TT	C	A	C	C	T	G	AGA	T	A	AAGA	T	A	C	A	C	C	G	G	0	0	2	f	
Hd1_25	AAT	C	C	k	G	G	T	C	A	G	A	A	A	CAG3	A	n	C	TT	C	T	T	C	C	G	AGA	T	A	AAGA	T	A	C	A	C	C	G	G	0	0	1	f	
Hd1_35	AAC	C	T	k	G	G	T	C	A	G	A	A	A	CAG3	A	n	C	TT	C	A	C	A	C	G	AGA	T	A	AAGA	T	A	C	A	C	C	G	G	0	0	1	f	
Hd1_36	AAC	C	T	k	G	G	T	C	A	G	A	A	A	CAG3	A	n	C	TT	C	A	C	C	C	G	AGA	T	A	AAGA	T	A	C	A	C	C	G	G	0	0	1	f	
Hd1_37	AAC	C	T	h	G	G	T	C	A	G	A	A	A	CAG3	A	n	C	TT	C	A	C	C	C	G	AGA	T	A	AAGA	T	A	C	A	C	C	G	G	0	0	1	f	
Hd1_38	AAC	C	T	h	G	G	T	C	A	G	A	A	A	CAG3	A	n	C	TT	C	A	C	C	C	G	AGA	T	A	AAGA	T	A	C	A	C	C	G	G	0	0	3	f	
Hd1_40	AAC	C	T	g	A	A	A	C	G	A	G	A	G	CAG3	A	n	C	TT	C	A	C	C	C	G	AGA	T	A	AAGA	T	A	C	A	C	C	G	G	0	0	1	f	
Hd1_41	AAC	C	T	g	A	A	A	C	G	A	G	A	G	9bp	A	n	C	TT	T	A	T	C	C	G	AGA	C	C	AAGA	C	G	C	G	A	G	G	G	0	0	1	f	
Hd1_42	AAC	C	T	i	A	A	A	C	G	A	G	A	G	CAG3	A	n	C	TT	T	A	T	C	C	G	AGA	C	C	AAGA	C	G	C	G	A	G	G	G	0	0	1	f	

D

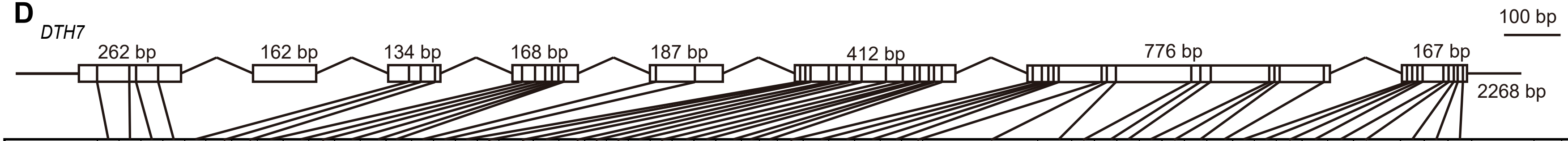

Position	47	125	140	204	477	506	547	584	616	641	664	667	680	728	840	881	933	934	953	1002	1016	1055	1147	1191	1221	1227	1259	1266	1287	1331	1369	1382	1385	1397	1515	1516	1546	1747	1768	1791	1948	1955	1967	2094	2110	2113	2129	2141	2220	2221	2260	2265	2267	No. of cvs			f / nf	Group
Position Variation	G16A	T42I	P47R	E68D	-	R169H	R183G	G195D	S206G	N214S	D222N	N223D	T227M	G243R	K280N	N294S	-	indel	R318K	-	R339Q	R352V	R383T	-	-	-	G420D	-	-	R444H	V457M	S461L	L462P	L466S	K505N	indel	indel	H583N	N590D	Q597H	G650R	I652T	E656G	-	Y704H	Stop	L710P	Q714R	-	indel	Q754N	R755S	Stop	I	J	W		
DTH7_2	G	T	C	T	C	G	C	G	A	A	G	G	T	C	G	A	C	TT	A	A	G	C	G	T	T	A	A	T	G	A	A	C	C	T	G	AACGTTGT	GA	C	A	A	G	T	G	C	T	C	T	A	G	39bp	G	A	G	1	1	0	f	groupA
DTH7_3	G	C	C	G	C	G	C	G	A	A	G	G	T	C	G	A	C	TT	G	A	G	C	G	T	T	G	G	C	G	A	A	C	T	G	G	AACGTTGT	GA	C	A	A	G	T	A	C	T	C	T	G	G	39bp	G	A	G	0	0	1	f	
DTH7_4	G	T	C	G	C	G	C	G	A	A	G	G	T	C	G	A	C	TT	G	A	G	C	G	T	T	A	G	C	G	A	A	C	T	G	G	AACGTTGT	GA	C	A	A	G	T	G	C	T	C	T	G	G	39bp	G	A	G	0	1	0	f	
DTH7_5	G	T	C	G	C	G	C	G	A	A	G	G	T	C	G	A	C	TT	G	A	G	C	G	T	T	A	G	C	G	A	A	C	T	G	G	AACGTTGT	GA	C	A	A	G	T	A	C	T	C	T	G	G	39bp	G	A	G	0	0	1	f	
DTH7_6	C	T	C	G	C	A	C	G	A	A	G	G	T	C	G	A	C	TT	G	A	G	C	G	T	T	A	G	C	G	A	A	C	T	G	G	AACGTTGT	GA	C	A	A	G	T	A	C	T	C	T	G	G	39bp	G	A	G	0	2	0	f	
DTH7_7	C	T	C	G	C	G	C	G	A	A	G	G	T	C	G	A	C	TT	G	A	G	C	G	T	T	A	G	C	G	A	A	C	T	G	G	AACGTTGT	GA	C	A	A	G	T	A	C	T	C	T	G	G	39bp	G	A	G	0	1	5	f	
DTH7_8	G	T	G	T	C	G	C	G	A	A	G	G	T	C	G	A	C	TT	G	A	G	C	G	T	T	A	G	C	G	A	A	C	T	G	G	AACGTTGT	GA	C	A	A	G	T	G	C	T	C	T	A	G	39bp	G	A	G	1	1	0	f	
DTH7_9	G	T	C	T	C	G	C	G	A	A	G	A	T	C	G	A	C	TT	G	A	G	C	G	T	T	A	G	C	G	A	A	C	T	G	G	AACGTTGT	GA	C	A	A	G	T	G	C	T	C	C	G	G	39bp	G	A	G	0	2	0	f	
DTH7_10	G	T	C	T	C	G	C	G	A	A	G	A	T	C	G	A	C	TT	G	A	G	C	G	T	T	A	G	C	G	A	A	C	T	G	G	AACGTTGT	GA	C	A	A	G	T	G	C	T	C	T	G	G	39bp	G	A	G	0	2	0	f	
DTH7_11	G	T	C	T	C	G	C	G	G	A	G	G	T	C	G	A	C	TT	G	A	G	C	G	T	T	A	G	C	G	A	A	C	T	G	G	AACGTTGT	GA	C	A	A	G	T	G	C	T	C	T	G	G	39bp	G	A	G	0	3	0	f	
DTH7_12	G	T	C	T	C	G	C	G	A	A	G	G	T	C	G	A	C	TT	G	A	G	C	G	T	T	A	G	C	G	A	A	C	T	G	G	AACGTTGT	GA	C	A	A	G	T	G	C	T	C	T	G	G	39bp	G	A	G	0	14	0	f	
DTH7_13	G	T	C	T	C	G	C	G	A	A	G	G	T	C	G	A	C	TT	G	A	G	C	G	T	T	A	G	C	G	A	A	C	T	G	G	AACGTTGT	2bp	C	A	A	G	T	G	C	T	C	T	G	G	39bp	G	A	G	0	1	0	f	
DTH7_14	G	T	G	T	C	G	C	G	A	A	G	G	T	C	G	A	C	TT	G	A	G	C	G	T	T	A	G	C	G	A	A	C	T	G	G	AACGTTGT	GA	C	A	A	G	T	G	C	T	C	T	G	G	39bp	G	A	G	0	1	0	f	
DTH7_15	G	T	C	T	C	G	C	G	A	A	G	G	T	C	G	A	C	TT	G	A	G	C	G	T	T	A	G	C	G	A	A	C	T	G	G	AACGTTGT	GA	C	A	A	G	T	G	C	T	C	T	A	G	39bp	G	A	G	1	0	0	f	
DTH7_16	G	T	C	T	C	G	C	G	A	A	G	A	T	C	G	A	C	TT	A	A	G	C	G	T	T	A	G	C	G	A	A	C	T	G	G	AACGTTGT	GA	C	A	A	G	T	G	C	T	C	T	G	G	39bp	G	A	G	0	2	0	f	
DTH7_17	G	T	C	T	C	G	C	G	A	A	G	G	T	C	G	A	C	TT	G	A	G	C	G	C	T	A	G	C	G	A	A	C	T	G	G	AACGTTGT	GA	C	A	A	G	T	G	C	T	C	T	G	G	39bp	G	A	G	1	1	0	f	
DTH7_18	G	T	C	T	C	G	C	G	A	A	G	G	T	C	G	A	C	TT	A	A	G	C	G	T	T	A	G	C	G	A	A	C	T	G	G	AACGTTGT	GA	C	A	A	G	T	G	C	T	C	T	A	G	39bp	G	A	G	1	0	0	f	
DTH7_19	G	T	G	T	C	G	C	G	A	A	G	G	T	C	G	A	C	TT	A	A	G	C	G	T	T	A	G	C	G	A	A	C	T	G	G	AACGTTGT	GA	C	A	A	G	T	G	C	T	C	T	G	G	39bp	G	A	G	0	1	0	f	
DTH7_20	G	T	C	T	C	G	C	G	A	A	G	G	T	C	G	A	C	TT	A	A	G	C	G	T	T	A	G	C	G	A	A	C	T	G	G	AACGTTGT	GA	C	A	A	G	T	G	C	T	C	T	G	G	39bp	G	A	G	0	3	0	f	
DTH7_1	G	C	C	G	T	G	T	G	A	G	G	G	C	G	G	A	T	2bp	G	A	G	C	G	T	T	A	G	T	G	G	G	C	C	G	G	AACGTTGT	GA	C	A	T	G	T	G	C	T	C	T	G	G	39bp	G	A	G	0	0	1	f	groupB
DTH7_21	G	C	C	G	C	G	G	A	A	G	G	G	C	C	G	A	C	TT	G	A	G	C	G	T	T	A	G	T	G	A	G	T	C	G	G	AACGTTGT	GA	C	A	T	C	C	G	C	T	C	T	G	G	39bp	G	A	G	0	0	1	f	
DTH7_22	G	C	C	G	C	G	C	A	A	G	G	G	C	C	G	A	C	TT	G	A	G	C	G	T	T	A	G	T	G	A	G	C	C	G	G	AACGTTGT	GA	C	A	T	C	C	G	C	T	C	T	G	G	39bp	G	A	G	0	0	5	f	
DTH7_23	G	C	C	G	C	G	C	G	A	G	A	G	T	C	G	A	C	TT	G	A	G	C	A	T	T	A	G	T	G	A	G	C	C	G	G	AACGTTGT	GA	C	A	A	G	T	G	C	T	C	T	G	A	39bp	A	C	A	0	0	2	nf	
DTH7_24	G	C	C	G	C	G	C	G	A	G	G	G	T	C	G	A	C	TT	G	A	G	C	A	T	T	A	G	T	G	A	G	C	C	G	G	AACGTTGT	GA	C	A	A	G	T	G	C	T	C	T	G	A	a	A	C	A	0	0	1	nf	
DTH7_25	G	C	C	G	C	G	C	G	A	G	G	G	T	C	G	A	C	TT	G	G	G	T	A	T	A	A	G	T	G	A	G	C	C	G	G	AACGTTGT	GA	C	A	A	G	T	G	C	T	C	T	G	G	b	G	A	G	1	1	0	f	
DTH7_26	G	C	C	G	C	G	C	G	A	G	G	G	T	C	G	G	C	TT	G	G	G	T	A	T	A	A	G	T	G	A	G	C	C	G	G	AACGTTGT	GA	C	A	A	G	T	G	T	T	C	T	G	G	39bp	G	A	G	1	0	0	f	
DTH7_27	G	C	C	G	C	G	C	G	A	G	G	G	T	C	G	A	C	TT	G	G	G	T	A	T	A	A	G	T	G	A	G	C	C	G	G	AACGTTGT	GA	C	A	A	G	T	G	T	T	C	T	G	G	39bp	G	A	G	2	6	5	f	
DTH7_28	G	C	C	G	C	G	C	G	A	G	G	G	T	C	G	A	C	TT	A	A	G	C	A	T	T	A	A	T	G	A	G	C	C	T	G	AACGTTGT	GA	C	A	A	G	T	G	C	T	C	T	G	G	39bp	G	A	G	5	0	0	f	
DTH7_29	G	C	C	G	C	G	C	G	A	G	G	G	T	C	G	A	C	TT	G	A	G	C	A	T	T	A	A	T	G	A	G	C	C	T	G	AACGTTGT	GA	C	A	A	G	T	G	C	T	C	T	G	G	39bp	G	A	G	7	1	3	f	
DTH7_30	G	C	C	G	C	G	C	G	A	A	G	G	T	C	G	A	C	TT	G	A	G	C	A	T	T	A	A	T	G	A	G	C	C	T	G	AACGTTGT	GA	C	A	A	G	T	G	C	T	C	T	G	G	39bp	G	A	G	0	0	1	f	
DTH7_31	G	C	C	G	C	G	C	G	A	G	G	G	T	C	G	A	C	TT	G	A	G	C	A	T	T	A	A	T	G	A	G	C	C	T	G	AACGTTGT	GA	C	A	A	G	T	G	C	T	T	T	G	G	39bp	G	A	G	1	0	0	nf	
DTH7_32	G	C	C	G	C	G	C	G	A	G	G	G	T	C	G	A	C	TT	G	A	G	C	A	T	T	A	A	T	G	A	G	C	C	T	T	8bp	GA	C	A	A	G	T	G	C	T	C	T	G	G	39bp	G	A	G	5	1	0	f	
DTH7_33	G	C	C	G	C	G	C	G	A	G	G	G	T	C	G	A	C	TT	A	A	G	C	A	T	T	G	G	T	C	A	G	C	C	G	G	AACGTTGT	GA	C	A	A	G	T	G	C	C	C	T	G	G	39bp	G	A	G	2	0	0	f	
DTH7_34	G	C	C	G	C	G	C	G	A	G	G	G	T	C	G	A	C	TT	G	A	G	C	A	T	T	G	G	T	C	A	G	C	C	G	G	AACGTTGT	GA	C	A	A	G	T	G	C	C	C	T	G	G	39bp	G	A	G	4	0	0	f	
DTH7_35	G	C	C	G	C	G	C	G	A	G	G	G	T	C	G	A	C	TT	G	A	G	C	A	T	T	G	G	T	C	A	G	C	C	G	G	AACGTTGT	GA	C	A	A	G	T	G	C	T	C	T	G	G	39bp	G	A	G	1	1	2	f	
DTH7_36	G	C	C	G	C	G	C	G	A	G	G	G	T	C	G	A	C	TT	A	A	G	C	A	T	T	G	G	T	C	A	G	C	C	G	G	AACGTTGT	GA	C	A	A	G	T	G	C	T	C	T	G	G	39bp	G	A	G	2	0	0	f	
DTH7_37	G	C	C	G	C	G	C	G	A	G	G	G	T	C	T	A	C	TT	G	A	A	C	A	T	T	A	G	T	G	A	G	C	C	G	G	AACGTTGT	GA	C	A	A	G	T	G	C	T	C	T	G	G	39bp	G	A	G	0	0	1	f	
DTH7_38	G	C	C	G	C	G	C	G	A	A	G	G	T	C	G	A	C	TT	G	A	G	C	A	T	T	A	G	T	G	A	G	C	C	G	G	AACGTTGT	GA	C	A	A	G	T	G	T	T	C	T	G	G	39bp	G	A	G	0	0	1	f	
DTH7_39	G	C	C	G	C	G	C	G	A	G	G	G	T	C	G	A	C	TT	G	A	G	C	A	T	T	A	G	T	G	A	G	C	C	G	G	AACGTTGT	GA	A	A	A	G	T	G	C	T	C	T	G	G	39bp	G	A	G	0	0	6	f	
DTH7_40	G	C	C	G	C	G	C	G	A	G	G	G	T	C	G	A	C	TT	G	A	G	C	A	T	T	A	G	T	G	A	G	C	C	G	G	AACGTTGT	GA	C	G	A	G	T	G	C	T	C	T	G	G	39bp	G	A	G	0	0	3	f	
DTH7_41	G	C	C	G	C	G	C	G	A	G	G	G	T	C	G	A	C	TT	G	A	G	C	A	T	T	A	G	T	G	A	G	C	C	G	G	AACGTTGT	GA	C	A	A	G	T	G	C	T	C	T	G	G	39bp	G	A	G	0	0	5	f	

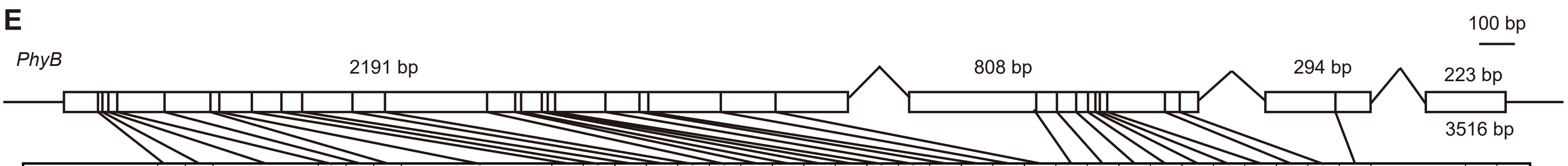

Position	96	104	124	150	282	412	436	525	605	663	808	899	1183	1263	1281	1338	1344	1368	1515	1606	1638	1833	1986	2546	2604	2658	2693	2714	2725	2745	2908	2947	3193	No. of cvs			f / nf	Group
Position variation	-	T35M	indel	-	-	E138K	indel	-	R202K	-	H270Y	S300N	A395S	-	-	-	-	-	E505D	P536S	-	-	-	N849I	-	K886N	T898M	E905V	A909T	-	D970N	Stop	V1065	I	J	W		
PhyB_2	C	C	GGCGGG	C	G	G	CTGTCGCCC	C	G	C	C	A	T	C	C	C	A	G	A	C	T	G	G	A	C	T	C	A	G	T	G	A	G	0	0	2	f	groupA
PhyB_3	C	C	GGCGGG	C	G	G	CTGTCGCCC	C	G	C	T	A	T	C	C	C	G	G	A	C	T	G	A	A	C	T	C	A	G	T	G	A	G	0	6	0	f	groupA
PhyB_4	C	C	GGCGGG	C	G	G	CTGTCGCCC	C	G	C	T	A	T	C	C	C	G	G	A	C	T	G	G	A	C	T	C	A	G	T	A	A	G	0	1	0	f	groupA
PhyB_5	C	C	GGCGGG	C	G	G	CTGTCGCCC	C	G	C	T	A	T	C	C	C	G	G	A	C	T	G	G	A	C	T	C	A	G	T	G	A	G	1	30	0	f	groupA
PhyB_7	C	C	GGCGGG	C	C	G	CTGTCGCCC	C	G	C	C	G	T	C	C	C	G	G	A	C	T	A	G	A	C	T	C	A	G	T	G	A	G	0	1	0	f	groupA
PhyB_28	C	C	GGCGGG	A	G	G	CTGTCGCCC	C	A	C	C	G	G	C	C	C	G	G	A	C	C	A	G	A	C	T	C	A	A	T	G	A	G	0	0	1	f	groupA
PhyB_1	G	C	GGCGGG	C	G	G	CTGTCGCCC	C	G	T	C	G	G	T	C	C	G	G	A	C	C	A	G	A	C	A	C	A	G	T	G	A	G	0	0	2	f	groupB
PhyB_6	C	C	GGCGGG	C	G	G	CTGTCGCCC	C	G	T	C	G	G	C	C	T	G	G	T	C	C	A	G	A	C	T	C	A	G	T	G	A	G	0	0	6	f	groupB
PhyB_8	C	C	GGCGGG	C	C	G	CTGTCGCCC	C	G	C	C	G	T	C	C	C	G	G	A	C	C	A	A	A	C	T	C	A	G	T	G	A	G	0	1	0	f	groupB
PhyB_9	C	C	GGCGGG	A	C	G	CTGTCGCCC	T	G	C	C	G	G	C	C	C	G	G	A	T	C	A	G	A	C	T	C	A	G	C	G	A	G	0	0	1	f	groupB
PhyB_10	C	C	GGCGGG	A	C	G	CTGTCGCCC	T	G	C	C	G	G	C	C	C	G	G	A	C	C	A	G	A	C	T	C	A	G	T	G	A	G	0	0	1	f	groupB
PhyB_11	C	C	GGCGGG	C	C	G	CTGTCGCCC	T	G	C	C	G	G	C	C	C	G	G	A	C	C	A	G	A	C	T	C	A	G	T	G	A	G	0	0	1	f	groupB
PhyB_12	C	C	GGCGGG	C	C	G	CTGTCGCCC	T	G	C	C	G	G	C	C	C	G	G	A	T	C	A	G	A	C	T	C	A	G	T	G	A	A	1	0	0	f	groupB
PhyB_13	C	C	GGCGGG	C	C	A	CTGTCGCCC	T	G	C	C	G	G	C	C	C	G	G	A	T	C	A	G	A	C	T	C	A	G	T	G	A	G	1	0	0	f	groupB
PhyB_14	C	C	GGCGGG	C	C	G	CTGTCGCCC	C	G	C	C	G	G	C	C	C	G	G	A	T	C	A	G	A	C	T	C	A	G	T	G	A	G	0	0	1	f	groupB
PhyB_15	C	C	GGCGGG	C	C	G	CTGTCGCCC	T	G	C	C	G	G	C	T	C	G	G	A	T	C	A	G	A	C	T	C	A	G	T	G	A	G	0	0	1	f	groupB
PhyB_16	C	C	GGCGGG	C	C	G	9bp	T	G	C	C	G	G	C	C	C	G	G	A	T	C	A	G	A	C	T	C	A	G	T	G	A	G	0	0	1	f	groupB
PhyB_17	C	C	GGCGGG	C	C	G	CTGTCGCCC	T	G	C	C	G	G	C	C	C	G	G	A	T	C	A	G	A	C	T	C	A	G	T	G	A	G	28	6	17	f	groupB
PhyB_18	C	C	GGCGGG	C	C	G	CTGTCGCCC	T	G	C	C	G	G	C	C	C	G	G	A	T	C	A	G	A	C	T	C	A	G	T	G	T	G	1	0	0	nf	groupB
PhyB_19	C	C	6bp	C	G	G	CTGTCGCCC	C	G	C	C	G	G	C	C	C	G	G	A	C	C	A	G	T	C	T	C	T	G	T	G	A	G	0	0	1	f	groupB
PhyB_20	C	C	6bp	C	G	G	CTGTCGCCC	C	G	C	C	G	G	C	C	C	G	G	A	C	C	A	G	A	C	T	T	T	G	T	G	A	G	0	0	1	f	groupB
PhyB_21	C	C	6bp	C	G	G	CTGTCGCCC	C	G	C	C	G	G	C	C	C	G	G	A	C	C	A	G	A	C	T	C	T	G	T	G	A	G	0	0	2	f	groupB
PhyB_22	C	C	GGCGGG	A	G	G	CTGTCGCCC	C	G	C	C	G	G	C	C	C	G	G	A	C	C	A	G	A	C	T	C	A	G	C	G	A	G	0	0	3	f	groupB
PhyB_23	C	C	GGCGGG	A	G	G	CTGTCGCCC	C	G	C	C	G	G	C	C	C	G	A	A	C	C	A	G	A	C	T	C	A	G	T	G	A	G	0	0	1	f	groupB
PhyB_24	C	T	GGCGGG	A	G	G	CTGTCGCCC	C	G	C	C	G	G	C	C	C	G	G	A	C	C	A	G	A	C	T	C	A	G	T	G	A	G	0	0	1	f	groupB
PhyB_25	C	C	GGCGGG	A	G	G	CTGTCGCCC	C	A	C	C	G	G	C	C	C	G	G	A	C	C	A	G	A	C	T	C	A	G	T	G	A	G	0	0	2	f	groupB
PhyB_26	C	C	GGCGGG	A	G	G	CTGTCGCCC	C	G	C	C	G	G	C	C	C	G	G	A	C	C	A	G	A	C	T	C	A	G	T	G	A	G	0	0	1	f	groupB
PhyB_27	C	C	GGCGGG	A	G	G	CTGTCGCCC	C	A	C	C	G	G	C	C	C	G	G	A	C	C	A	G	A	T	T	C	A	A	T	G	A	G	0	0	1	f	groupB

F

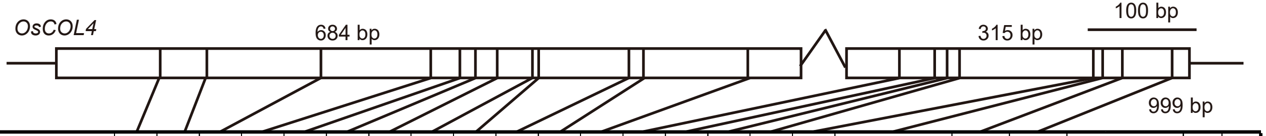

Position	96	138	243	344	370	385	404	438	442	526	540	634	733	765	777	788	911	919	938	984	No.of cvs			f / nf	Group
Position Variation	-	-	-	A115V	A124T	P129T	A135E	-	D148N	D176Y	-	N212D	A272S	-	K286N	E290A	T331N	indel	A340E	-					
OsCOL4_16	C	G	C	C	G	C	C	T	G	G	C	G	G	G	G	C	C	GCCGAC	C	C	0	1	1	f	groupA
OsCOL4_17	C	G	C	C	A	C	C	T	G	G	C	G	T	G	G	C	C	GCCGAC	C	C	0	1	0	f	
OsCOL4_18	C	G	C	C	G	C	C	T	G	G	C	G	T	G	G	C	C	GCCGAC	C	C	1	26	3	f	
OsCOL4_1	T	G	C	C	G	C	C	C	G	G	C	A	G	G	G	A	C	GCCGAC	C	C	0	0	5	f	groupB
OsCOL4_2	C	A	C	C	G	A	C	C	G	G	C	G	G	G	G	C	C	GCCGAC	C	C	7	1	5	f	
OsCOL4_3	C	G	C	C	G	A	C	C	G	G	C	G	G	C	G	C	C	GCCGAC	C	C	2	0	1	f	
OsCOL4_4	C	G	C	C	G	A	C	C	G	G	C	G	G	C	G	C	C	GCCGAC	A	C	0	0	1	f	
OsCOL4_5	C	G	C	T	G	A	C	C	G	G	C	G	T	G	G	C	C	GCCGAC	C	C	0	0	1	f	
OsCOL4_6	C	G	C	T	G	A	C	C	G	G	C	G	G	G	C	C	C	GCCGAC	C	C	8	7	4	f	
OsCOL4_7	C	G	C	T	G	A	C	C	G	G	C	G	G	G	G	C	C	GCCGAC	C	C	2	0	0	f	
OsCOL4_8	C	G	C	C	G	C	A	C	A	G	C	G	G	G	G	C	C	GCCGAC	C	C	0	0	1	f	
OsCOL4_9	C	G	A	C	G	C	C	C	G	G	A	G	G	G	G	C	C	GCCGAC	C	C	0	0	1	f	
OsCOL4_10	C	G	A	C	G	C	C	C	G	G	C	G	G	G	G	C	C	GCCGAC	C	G	0	0	5	f	
OsCOL4_11	C	G	A	C	G	C	C	C	G	G	C	G	G	G	G	C	C	GCCGAC	C	C	6	2	5	f	
OsCOL4_12	C	G	A	C	G	C	C	C	G	G	C	G	G	G	G	C	C	6bp	C	C	0	0	2	f	
OsCOL4_13	C	G	C	C	G	C	C	C	G	G	C	G	G	G	G	C	C	GCCGAC	C	C	2	1	18	f	
OsCOL4_14	C	G	C	C	G	C	A	C	G	G	C	G	G	G	G	C	C	GCCGAC	C	C	0	0	2	f	
OsCOL4_15	C	G	C	C	G	A	C	C	G	G	C	G	G	G	G	C	A	GCCGAC	C	C	1	0	0	f	
OsCOL4_19	C	G	C	C	G	C	C	C	G	T	C	G	G	G	G	C	C	GCCGAC	C	C	0	0	1	f	
OsCOL4_20	C	A	C	C	G	C	C	C	G	G	C	G	G	G	G	C	C	GCCGAC	C	C	0	0	1	f	
OsCOL4_21	C	G	C	C	G	A	C	C	G	G	C	G	G	G	G	C	C	GCCGAC	C	C	2	0	1	f	
OsCOL4_22	C	G	C	C	G	C	C	C	G	G	C	G	G	G	G	C	C	GCCGAC	C	G	0	0	1	f	

G

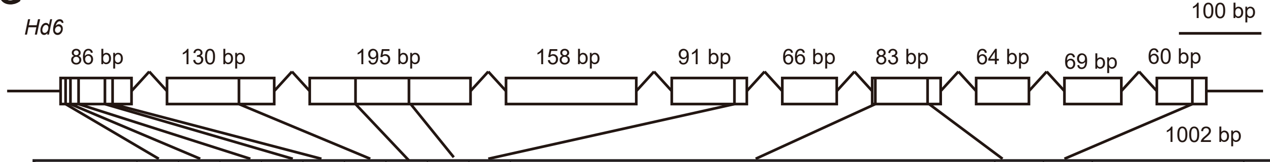

Position	6	11	22	55	64	176	271	336	645	727	792	986	No. of cvs			f / nf
Position Variation	-	A4G	A8P	W19R	E22K	K59R	Stop	-	-	indel	-	R329T	I	J	W	
Hd6_1	G	C	G	T	G	A	A	C	C	GTACTTGGAACAGAAGCACTAAATGCTTA	T	G	35	30	42	f
Hd6_2	G	C	G	T	G	A	A	C	C	GTACTTGGAACAGAAGCACTAAATGCTTA	T	C	0	0	1	f
Hd6_3	G	C	G	T	G	A	A	C	T	GTACTTGGAACAGAAGCACTAAATGCTTA	T	G	0	0	1	f
Hd6_4	A	G	C	T	A	A	A	C	C	GTACTTGGAACAGAAGCACTAAATGCTTA	T	G	0	0	1	f
Hd6_5	G	C	G	T	G	G	A	C	C	GTACTTGGAACAGAAGCACTAAATGCTTA	T	G	0	0	1	f
Hd6_6	G	C	C	T	G	A	A	C	C	GTACTTGGAACAGAAGCACTAAATGCTTA	T	G	0	0	1	f
Hd6_7	G	C	G	T	G	A	A	T	C	GTACTTGGAACAGAAGCACTAAATGCTTA	T	G	0	0	2	f
Hd6_8	G	G	C	T	A	A	A	C	C	GTACTTGGAACAGAAGCACTAAATGCTTA	T	G	0	0	3	f
Hd6_9	A	C	C	T	A	A	A	C	C	GTACTTGGAACAGAAGCACTAAATGCTTA	T	G	0	0	1	f
Hd6_10	G	C	G	C	G	A	A	C	C	GTACTTGGAACAGAAGCACTAAATGCTTA	T	G	0	2	0	f
Hd6_11	G	C	G	T	G	A	A	C	C	GTACTTGGAACAGAAGCACTAAATGCTTA	C	G	0	3	0	f
Hd6_12	G	C	G	T	G	A	T	C	C	GTACTTGGAACAGAAGCACTAAATGCTTA	T	G	0	9	0	nf
Hd6_13	G	C	G	T	G	A	A	C	C	29bp	T	G	0	1	0	f

H

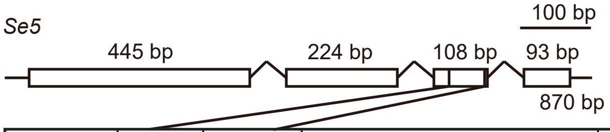

Position	700	771	No. of cvs			f / nf
Position Variation	-	-	I	J	W	
Se5_1	T	C	9	1	9	f
Se5_2	C	A	0	0	1	f
Se5_3	C	C	25	44	51	f
